# On the ability of the LR method to detect bias when there is pedigree misspecification and lack of connectedness

**DOI:** 10.1186/s12711-024-00943-1

**Published:** 2024-11-21

**Authors:** Alan M. Pardo, Andres Legarra, Zulma G. Vitezica, Natalia S. Forneris, Daniel O. Maizon, Sebastián Munilla

**Affiliations:** 1 Estación Experimental Agropecuaria Balcarce, Instituto Nacional de Tecnología Agropecuaria (INTA), B7620 Balcarce, Argentina; 2 Facultad de Ciencias Agrarias, Universidad Nacional de Mar del Plata, B7620 Balcarce, Argentina; 3 Council On Dairy Cattle Breeding, Bowie, MD USA; 4 INPT/INRAE-UMR 1388 GenPhySE, Toulouse, France; 5 Facultad de Agronomía, Universidad de Buenos Aires, C1417DSQ Buenos Aires, Argentina; 6 Instituto de Investigaciones en Producción Animal (INPA), CONICET-Universidad de Buenos Aires, C1427CWO Buenos Aires, Argentina; 7 Estación Experimental Agropecuaria Anguil, Instituto Nacional de Tecnología Agropecuaria (INTA), L6326 Anguil, Argentina

## Abstract

**Background:**

Cross-validation techniques in genetic evaluations encounter limitations due to the unobservable nature of breeding values and the challenge of validating estimated breeding values (EBVs) against pre-corrected phenotypes, challenges which the Linear Regression (LR) method addresses as an alternative. Furthermore, beef cattle genetic evaluation programs confront challenges with connectedness among herds and pedigree errors. The objective of this work was to evaluate the LR method's performance under pedigree errors and weak connectedness typical in beef cattle genetic evaluations, through simulation.

**Methods:**

We simulated a beef cattle population resembling the Argentinean Brangus, including a quantitative trait selected over six pseudo-generations with a heritability of 0.4. This study considered various scenarios, including: 25% and 40% pedigree errors (PE-25 and PE-40), weak and strong connectedness among herds (WCO and SCO, respectively), and a benchmark scenario (BEN) with complete pedigree and optimal herd connections.

**Results:**

Over six pseudo-generations of selection, genetic gain was simulated to be under- and over-estimated in PE-40 and WCO, respectively, contrary to the BEN scenario which was unbiased. In genetic evaluations with PE-25 and PE-40, true biases of − 0.13 and − 0.18 genetic standard deviations were simulated, respectively. In the BEN scenario, the LR method accurately estimated bias, however, in PE-25 and PE-40 scenarios, it overestimated biases by 0.17 and 0.25 genetic standard deviations, respectively. In herds facing WCO, significant true bias due to confounding environmental and genetic effects was simulated, and the corresponding LR statistic failed to accurately estimate the magnitude and direction of this bias. On average, true dispersion values were close to one for BEN, PE-40, SCO and WCO, showing no significant inflation or deflation, and the values were accurately estimated by LR. However, PE-25 exhibited inflation of EBVs and was slightly underestimated by LR. Accuracies and reliabilities showed good agreement between true and LR estimated values for the scenarios evaluated.

**Conclusions:**

The LR method demonstrated limitations in identifying biases induced by incomplete pedigrees, including scenarios with as much as 40% pedigree errors, or lack of connectedness, but it was effective in assessing dispersion, and population accuracies and reliabilities even in the challenging scenarios addressed.

**Supplementary Information:**

The online version contains supplementary material available at 10.1186/s12711-024-00943-1.

## Background

The main objective of a genetic evaluation program is to estimate the genetic merit of the selection candidates and, ultimately, to predict the future performance of their progeny. Accordingly, checking the quality of these predictions is an important way to validate them. Results of a genetic evaluation program (i.e. estimated breeding values, EBVs) can be verified by cross-validation techniques, which have become more relevant in recent years due to the availability of genome-wide markers in animals and plants [1–3].

Common validation methods involve assessing the model’s predictive performance (e.g. *predictive ability*) using cross-validation approaches, which entail dividing the whole data into *training* set(s) (data on which the model is fitted) and *validation* set(s) (data against which the model predictions are tested), often at random. However, there are some important issues regarding cross-validations in animal breeding as the prediction target, the breeding value, is not observable and consequently EBVs must be validated against pre-corrected phenotypes. Legarra and Reverter [4] pointed out that cross-validation techniques based on pre-corrected phenotypes can be difficult to implement in genetic evaluation programs due to several reasons: (1) limited number of parents and families in the pedigrees; (2) incorrect estimates of the pre-corrected data using fixed effects solutions; (3) not feasible for indirectly observed traits (e.g. maternal) or traits that are complex to model (e.g. scores). Additionally, data truncation should be time-based (e.g., based on a cut-off date) to align with common practices in breeding programs for predicting and using EBVs.

Legarra and Reverter [5] developed a validation method called LR method (acronym for *Linear Regression method*). LR method compares the EBVs of a same group of *focal* (or target) individuals estimated from two datasets: a reduced or *partial* dataset ($EB{V}_{p}$) and a *whole* dataset ($EB{V}_{w}$) which contains all “partial” records plus more recent ones. More specifically, the validation procedure is carried out by comparing the estimated values of a series of statistics calculated from the two sets of EBVs against their expected values. These expected values were derived assuming that *Best Linear Unbiased Prediction* (BLUP) theory assumptions are fulfilled (see Legarra and Reverter [5] and Macedo et al. [6] for further methodological details). Recently, the applicability of the LR method has been successfully extended to predictions based on conditional means [7, 8]. Similar methods in which “early” and “late” evaluations are compared are used or proposed ([9, 10], respectively) in the Interbull validation tests, although these methods are more tailored to dairy cattle scenarios.

Beef cattle genetic evaluation programs face significant challenges in controlling inconsistencies in their results due to the sometimes-poor quality of both phenotypic and pedigree records. Typically, these undesirable effects occur due to two main factors: incorrect or unknown pedigree information, resulting in the use of an inaccurate relationship matrix [11], and weak connectedness among herds [12, 13]. Incorrect or incomplete pedigree recording, especially in scenarios involving multiple-sire mating, can potentially lead to biased EBVs [14, 15]. Meanwhile, lack of connectedness is particularly important in beef cattle where the level of AI adoption is much lower than in dairy cattle [16].

It is unclear whether the undesirable effects of pedigree misspecification and lack of connections can be detected with the Linear Regression method, which assumes and has been tested under the hypothesis, that the correct pedigree is known (e.g. [6, 17]). In this study, we evaluated by simulation the performance of the LR method under scenarios of weak connectedness and pedigree errors.

## Methods

### Simulation

**Fig. 1:**
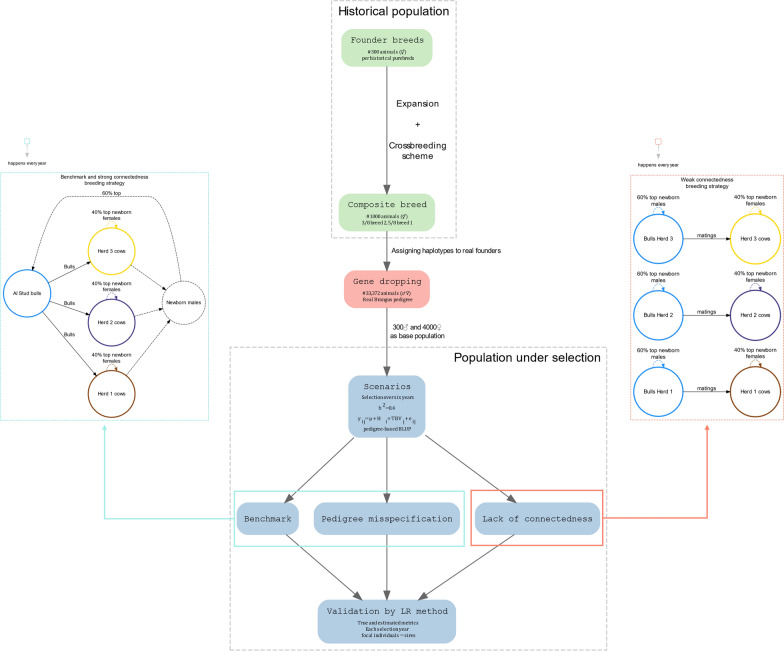
An overview of the whole simulation process. We show a flowchart of the simulation steps: create historical haplotype sequences for two founder breeds, recombine and crossbreed to achieve a composite population, allocate haplotypes to real Brangus pedigree founders, perform gene dropping, select base population and simulate TBV, explore three scenarios (benchmark, pedigree errors, lack of connectedness) over six years of selection, and summarize LR method metrics

We simulated a beef cattle population mimicking an established composite breed inspired by the Argentinean Brangus (37.5% *B. taurus indicus* and 62.5% *B. taurus taurus*). The simulation involved three steps (Fig. 1). First, we created a historical population based on the genomic architecture parameters for a composite breed. Next, we took individuals from this historical population as founders of a gene-dropping procedure using the pedigree of the Argentinean Brangus population to simulate new generations of individuals. Finally, we simulated a quantitative trait and applied selection on it along six more generations. The whole simulation process was replicated 20 times for each scenario. We describe hereafter the simulation in more detail.

#### Historical population

##### Founder breeds

We initiated the simulation with demographic parameters of cattle [18] via the Markovian Coalescent Simulator MaCS [19] implemented in AlphaSimR [20]. A divergence event was included 50,000 generations ago to produce two breeds that are genetically sufficiently different (i.e., mirroring the subspecies *B. taurus taurus* and *B. taurus indicus*). Next, a population expansion was simulated over six generations through random matings conducted independently within each subpopulation. At the end of the sixth generation of expansion, 24,000 animals were available in each subpopulation and these animals were then used for the subsequent crossbreeding scheme. The simulated genomes in each breed had 29 autosomal chromosomes, each with 10^8^ base pairs. The mutation rate was set to 2.5e−8 per base pair per generation. We kept 3500 randomly chosen segregating sites per chromosome, summing up to a total 101,500 polymorphic loci.

##### Creation of the composite breed

Using individuals from the previously generated breeds, a crossbreeding scheme of four generations was simulated to establish a composite breed with breed proportions typical of Brangus cattle ($3/8$ Brahman and $5/8$ Angus). The *F*_1_ population was originated from the random mating of 50 randomly sampled males from *breed 1* with 50 randomly sampled females from *breed 2*. The 50 *F*_1_ females thus created were next randomly mated with 50 randomly sampled males from *breed 2* to generate 50 *backcross* individuals. Then, these 50 *backcross* individuals were used as males and mated with 1000 females from *breed 1*. After this mating scheme, 1000 individuals were obtained with breed proportions of $3/8$ from *breed 2* and $5/8$ from *breed 1*. These individuals constituted the pool of founders for the gene-dropping procedure on the real pedigree that is described below.

#### Gene dropping

A real pedigree of 530,363 animals was obtained from the Brangus Genetic Evaluation Program in Argentina (www.brangus.org.ar/programa-erbra/; accessed 20 August 2022). This pedigree was pruned to include the last two cohorts of calves and all their ancestors using the prune “Pedigree” option of RelaX2 software [21]. The final pedigree comprised a total of 33,372 animals, born between 1920 and 2022.

The gene-dropping procedure started by randomly assigning haplotypes from the simulated pool of composite individuals to each of the founders of the real pedigree. Then, the genomes of all descendants were generated by randomly dropping these founder haplotypes through the real Brangus pedigree. The gene dropping step was used to replicate the complex patterns of linkage disequilibrium (LD) observed in the current Argentinean Brangus population pedigree. Statistics of LD showed good agreement between simulated genomes and observed LD patterns in the actual Brangus breed (not shown).

#### Population under selection

The composite breed population was next subjected to a selection process along six overlapping pseudo-generations (loosely called “years” in what follows). To do so, we started by randomly choosing 4300 individuals (300 males and 4000 females) from the last generation of the gene-dropping stage as the base population (year 0) and simulated their true breeding values (TBV) for a purely additive trait of moderate heritability (0.4). Breeding values were based on randomly taking 10,000 segregating sites as quantitative trait loci (QTL) and sampling their allele substitution effects from a Gamma distribution (shape parameter equal to 0.4), resulting in an additive genetic variance of 0.4.

Phenotypes, ${y}_{ij}$, in any year were simulated as follows:

$${y}_{ij}=\mu +{H}_{i}+TBV_{j}+{e}_{ij},$$

where $\mu$ is an overall mean, ${H}_{i}$ is the effect for herd $i$, $TB{V}_{j}$ $\sim N(0,{\sigma }_{a}^{2})$ is the true breeding value of animal $j$ with ${\sigma }_{a}^{2}=0.4$ and ${e}_{ij}\sim N(0,{\sigma }_{e}^{2})$ is a random error effect with ${\sigma }_{e}^{2}=0.6$. In addition, each individual was assigned to one out of three herds; specifically, it was assigned to the herd of its dam. As it is explained in the next section, the assignments to the herds in the base population were carried out either at random or in an oriented manner, depending on the scenario.

Each year, a pedigree-based BLUP model was fitted on the available data and selection was practiced based on the estimated breeding values (EBVs). Depending on the simulated scenario, the model included either a general mean (for pedigree-error comparisons) or both, a general mean and a herd-year effect (for connectedness comparisons). EBVs were obtained using the BLUPF90 family of programs [22]. Forty percent of the cows with the lowest EBVs were replaced by the top newborn females, selected within the herd (*N* ≈ 533 females). In addition, 60% of the bulls with the lowest EBVs were replaced by new-born males, while replacements were chosen according to the scenario evaluated, ranging from a common pool to within herd (*N* = 180 to *N* = 60 bulls, respectively). On average there were approximately 1333 cows per herd per year. After the last generation, the pedigree had 28,300 animals with records (4300 from the base or year 0, plus 4000 progenies born in each of the six years) for each of the scenarios and replicates evaluated. The final number of sires and dams in the pedigree depended on the simulated scenarios and replicates, ranging from 1199 to 1200 sires and from 10,673 to 12,000 dams.

### Scenarios evaluated

We explored three different scenarios: (1) a benchmark scenario with an ‘ideal’ population structure for a genetic evaluation program, including strong connections between herds and a complete pedigree; (2) a scenario with typical errors in pedigree specification commonly found in beef cattle populations; and (3) a scenario with a population structure that exhibits severe connectedness problems.

#### Benchmark

In the “ideal” or benchmark scenario (BEN), male replacements were randomly chosen from a common pool of bulls, mimicking an AI stud. According to Selle et al. [23] and Powell et al. [24] this strategy guarantees good connectedness between herds and years by using these bulls as reference sires and helps in achieving equally related animals within and between herds. The pool was refreshed every year with newborn males from the top 60% EBVs across herds, replacing the bottom 60% of bulls. Males and females of the base population were allocated at random to the herds. After six years of selection, there were 1200 sires and 12,000 dams in the pedigree file. In this scenario, simulated herd effects were set to zero and not included in the evaluation model.

#### Pedigree misspecification

The population structure and breeding strategy (both for males and females) were the same as in the BEN scenario. However, each year we generated errors in the pedigree at two levels. At first, we chose at random a percentage of animals (25% and 40%; PE-25 and PE-40, respectively), and we assigned to half of them parents unknown in the pedigree file used for running the genetic evaluation. Second, for the other half of the animals chosen, we assigned a wrong sire, which was selected from the same generation as the true one. Although estimates of error rates in real pedigrees vary widely by country and breed [25], we consider PE-25 and PE-40 to represent high and extreme rates, respectively.

In this scenario, simulated herd effects were set to zero as in BEN and not included in the model. This strategy ensured that the observed effects of pedigree errors were not affected by issues related to estimation of the herd effects.

#### Lack of connectedness

**Fig. 2:**
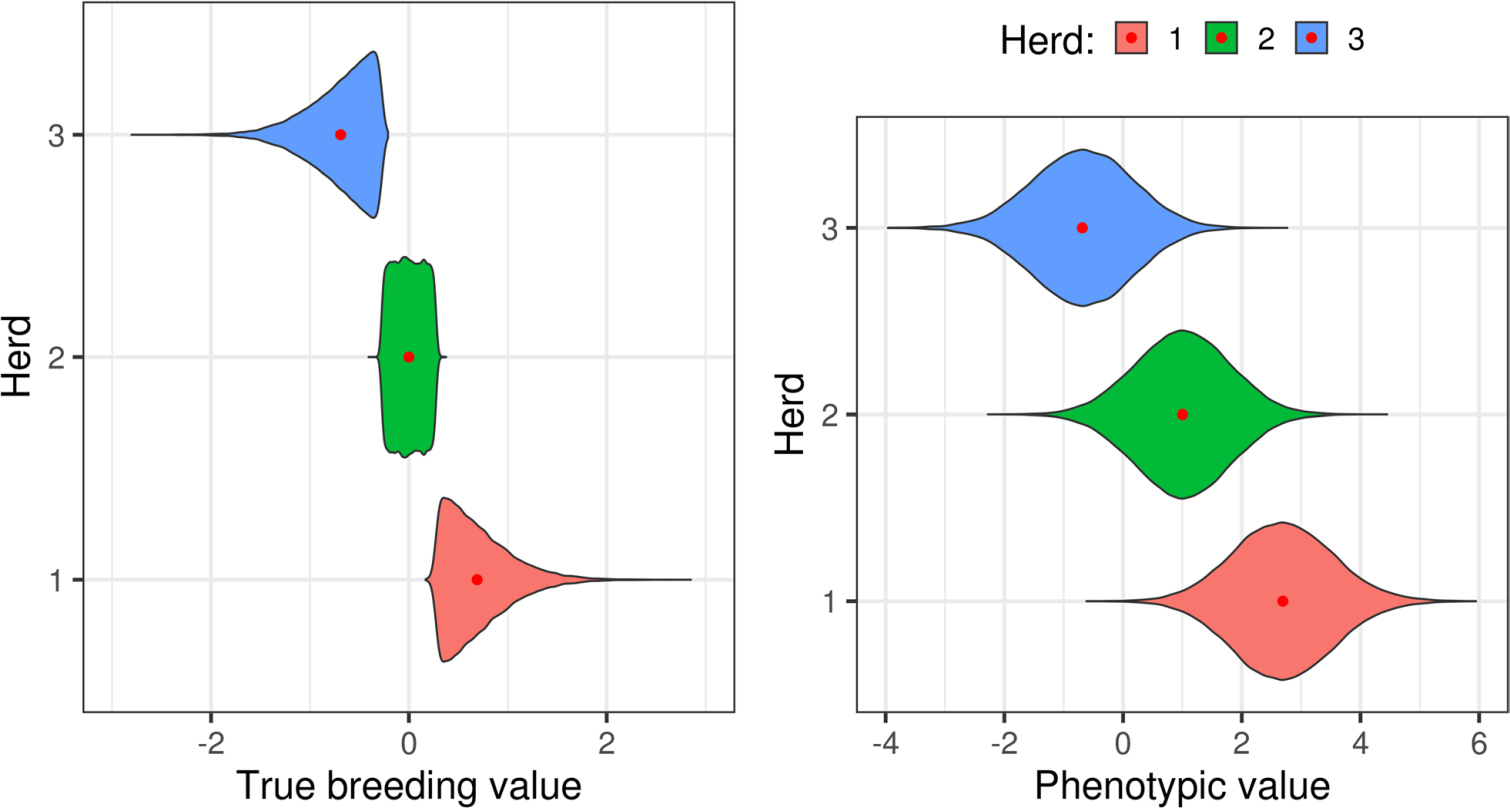
True breeding values and phenotypic values across herds in the base population under weak connectedness. Red dots denote means

To simulate problems due to lack of connectedness, males and females of the base population were arbitrarily allocated to each herd according to their TBV: the best $1/3$ of the animals were assigned to herd 1, the following $1/3$ to herd 2, and the worst $1/3$ to herd 3 (Fig. 2). This situation can be found e.g. when one country is producing the genetic improvement and the other are importers, which introduce genetic material without the information on which selection was based. Additionally, when simulating the phenotypes, we set herd effects to the values *H*_1_ = 2, *H*_2_ = 1, and *H*_3_ = 0, respectively, starting from the base population and in subsequent years. Therefore, by design, genetic level and herd effects were confounded in the base population, and herds with best management had best genetics too. This setup was chosen to induce true bias due to weak connectedness. A simpler design based on randomly assigning the founders into different herds and then creating different connectedness levels will produce no true bias [13].

Then, two different levels of connectedness were generated. First, and in order to achieve a scenario with very weak connectedness (WCO), in subsequent years, males’ replacements were selected from within herd, which resulted, after six years, in animals that were strongly related within herd but genetically distant between herds. Second, to achieve a scenario with stronger connectedness (SCO), a small modification was introduced in the way males were replaced. After an initial service in the herd to which they were originally assigned, the bulls were brought together into a common pool, and from that point on, replacements were selected in the same way as in the BEN scenario.

One important concern regarding the strategy we used to simulate the connectedness scenarios was whether strong and weak connections between herds were indeed achieved. We address this issue in a supplementary material section (see Additional file 1), where we describe in detail the measures we used to verify that our scenarios were truly contrasting in terms of connectedness. The results of these analyses are further visualized in Additional file 2 Figures S1 to S5. Overall, the results indicate that the SCO scenario achieved a significantly higher level of connectedness than WCO, particularly between herds in the final years of selection. Furthermore, the patterns observed in genomic relationships among animals were consistent with the expected outcomes of each scenario, highlighting the impact of the different connectedness strategies on genetic distance.

In both the WCO and SCO scenarios, true simulated effects for the three herds were compared to their estimated effects ($\widehat{{H}_{1}}$, $\widehat{{H}_{2}}$ and $\widehat{{H}_{3}}$, respectively) using the herd-year effects solutions (best linear unbiased estimates; BLUE). For comparison, we compute averages and 95% confidence intervals (%CI) for the BLUEs generated in the last genetic evaluation executed, across the 20 replicates, with the 95%CI calculated using the standard error of the mean across replicates.

### Validation by means of the LR method

#### Linear regression (LR) method

The LR method is based on statistics that compare the EBVs of a target group, called *focal* individuals, estimated from a *partial* dataset and a *whole* dataset. The latter contains all partial records plus more recent ones. These statistics are estimators of the population measures of bias and dispersion of EBV, or they estimate functions of the population accuracies (see Legarra and Reverter [5], for details). Let ${\widehat{a}}_{p}$ and ${\widehat{a}}_{w}$ represent the estimated breeding values of the focal individuals obtained from the partial and the whole datasets, respectively. Then, the statistics proposed by Legarra and Reverter [5] are:

- ${\widehat{\Delta }}_{p}=\overline{{\widehat{a}}_{p}}-\overline{{\widehat{a}}_{w}}$, which estimates the population *bias* and has an expected value of zero if the evaluation is unbiased.
- ${\widehat{b}}_{p}=cov({\widehat{a}}_{w},{\widehat{a}}_{p})/var({\widehat{a}}_{p})$, which estimates the population *dispersion* of EBV and has an expectation equal to one if there is no over/under dispersion;
- ${\widehat{\rho }}_{w,p}=cor({\widehat{a}}_{p},{\widehat{a}}_{w})$, which estimates the *ratio of population accuracies*, and it is a direct estimator of relative increase of accuracy from partial ($ac{c}_{p}$) to whole ($ac{c}_{w}$). This statistic has expected value $ac{c}_{p}/ac{c}_{w}$;
- $\widehat{{\rho }_{w,p}^{2}}=cov({\widehat{a}}_{w},{\widehat{a}}_{p})/var({\widehat{a}}_{w})$, which estimates the *ratio of population reliabilities*. It has expected value of $ac{c}_{p}^{2}/ac{c}_{w}^{2}$ and is proportional to the relative gain in average reliabilities as new information is added. As in the ${\widehat{\rho }}_{w,p}$ estimator, a high value of $\widehat{{\rho }_{w,p}^{2}}$ means a small increase in population reliability, whereas a low value means a large increase in population reliability, when we move from *partial* to *whole* dataset.
- $\widehat{ac{c}_{p}^{2}}=cov({\widehat{a}}_{p},{\widehat{a}}_{w})/{\sigma }_{a*}^{2}$, which estimates the *absolute selected reliability*. The term in the denominator of this metric (${\sigma }_{a*}^{2}$) refers to the additive genetic variance of the *focal* individuals (that we knew from simulations). This metric gives the “selected” reliability, i.e., it does not estimate model-based reliability from the mixed-model equations (MME) [26];
- ${\widehat{rel}}_{p}=1-{\sigma }_{a*}^{2}/{\sigma }_{a}^{2}(1-{\widehat{acc}}_{p}^{2})$, which estimates the *absolute unselected reliability* as if there was no selection and the result matches with theoretical reliabilities from the inverse of the MME [27]. The term in the denominator of this metric (${\sigma }_{a}^{2}$) refers to the additive genetic variance of the base population (that we knew from simulations).

Of the last two statistics related to “absolute” reliabilities, we only report results from the last one (${\widehat{rel}}_{p}$). Notice that both measures are linearly related and thus proportional to each other. Of both, ${\widehat{rel}}_{p}$ estimates values within the range of classical reliabilities from MME, providing a comprehensive metric for evaluating genetic evaluation methods under varying conditions of pedigree accuracy and connectedness.

#### Defining whole and partial data sets for focal individuals

In this study, we defined the focal individuals to be the selected sires. We compared their “partial” EBVs, ${\widehat{a}}_{p}$, based on a dataset where only their own record was available, with their “whole” EBVs, ${\widehat{a}}_{w}$, where now the dataset included also their offspring records. We carried out several comparisons, each taking ${\widehat{a}}_{p}$ from bulls born either in year ${n}_{p} ({n}_{p}=\left\{3, 4, 5\right\})$ and ${\widehat{a}}_{w}$ from years ${n}_{w}={n}_{p}+1 ({n}_{w}=\left\{4, 5, 6\right\})$: ${\widehat{a}}_{p3}$ vs ${\widehat{a}}_{w4}$, ${\widehat{a}}_{p3}$ vs ${\widehat{a}}_{w5}$, ${\widehat{a}}_{p3}$ vs ${\widehat{a}}_{w6}$, ${\widehat{a}}_{p4}$ vs ${\widehat{a}}_{w5}$, ${\widehat{a}}_{p4}$ vs ${\widehat{a}}_{w6}$, and ${\widehat{a}}_{p5}$ vs ${\widehat{a}}_{w6}$. From each of these comparisons, we obtained an estimate of each of the LR metrics and then averaged them out following the procedure described by Macedo et al. [27] to account for the overrepresentation of some of the years. For example, breeding values of bulls born in year *np* = 3 contribute to three out of six comparisons, whereas breeding values of bulls from year *np* = 5 contribute only to one comparison. Consequently, raw averages are inappropriate. As Macedo et al. [27] state, their procedure produces an estimate “as if the design was balanced”. In the Appendix we describe the procedure in more detail.

The “true” LR method metrics were obtained by comparing the EBVs in genetic evaluation at year $n$ (${\widehat{a}}_{p}$) with the corresponding TBV ($a$). For example, to compute the true bias and dispersion: ${\Delta }_{p}=\overline{{\widehat{a}}_{p}}-\overline{a}$ and ${b}_{p}=cov(a,{\widehat{a}}_{p})/var({\widehat{a}}_{p})$, respectively.

For comparisons involving connectedness scenarios, the focal bulls were grouped according to the herd where they were born. Thus, we obtained three estimates (one for each herd) for each evaluated statistic. Instead, the focal individuals for BEN, PE-25 and PE-40 scenarios were selected bulls indistinctly across the three herds. Importantly, to compute all statistics we always referred EBVs and TBVs to the same base generation. To do so, we subtracted the mean EBV of animals from the founder population (i.e., animals with both parents unknown and born in year “0”) from the EBV. Notice that in the PE-25 and PE-40 scenarios there are non-founder animals with both parents unknown.

When evaluating the performance of the LR method statistics, we report the mean and the standard deviation (SD) of true and estimated values, along with the Pearson correlations between them. Mean and SD values evaluate the LR method as a reliable estimator, while correlations evaluate the agreement between the estimated and true values.

## Results

In this section, we first briefly present the genetic gains obtained over the six years of selection, and then we address the results obtained regarding the performance of the LR method in the different scenarios tested.

### Genetic gains

**Fig. 3:**
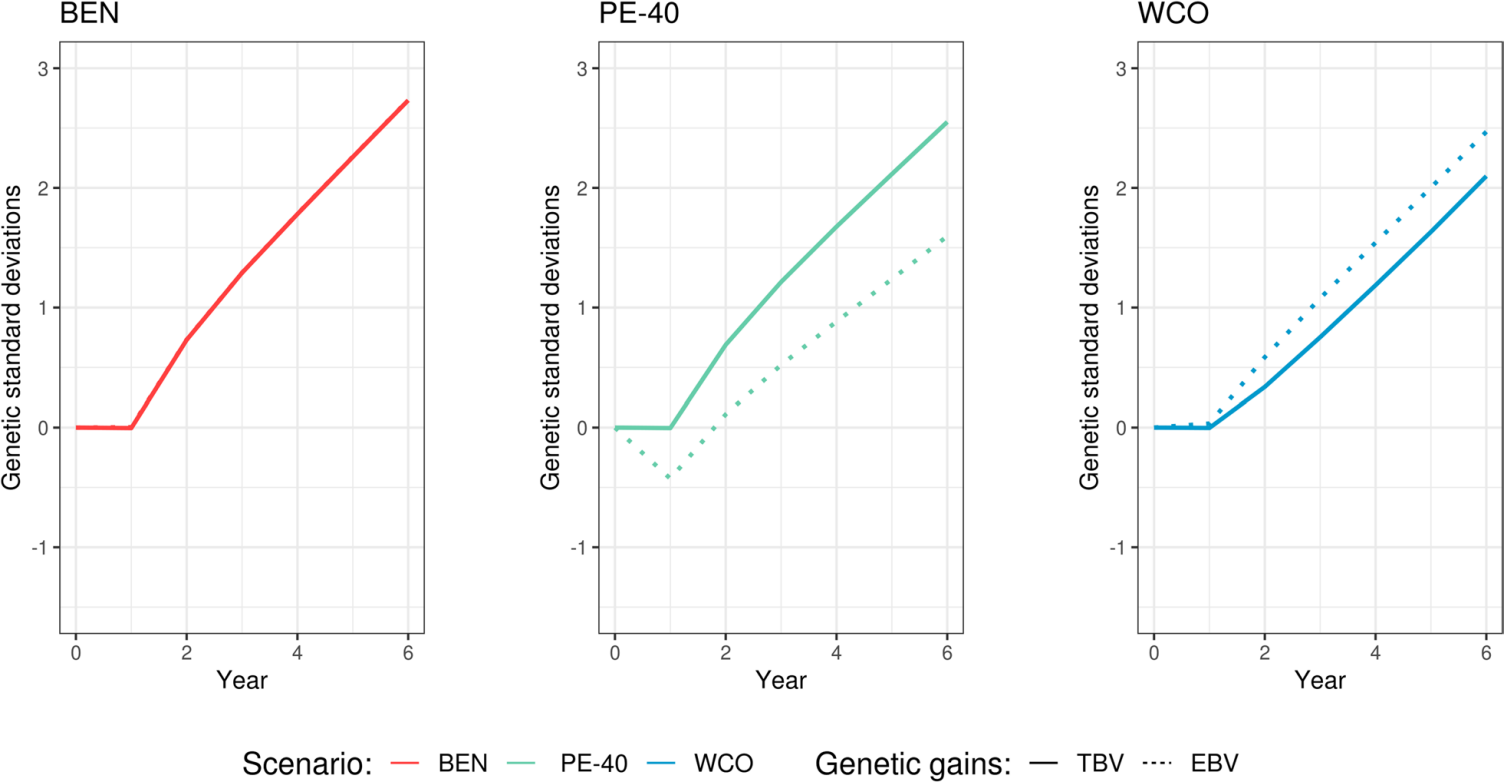
Genetic gains for the extreme simulated scenarios. The plots illustrate the changes in true (solid line) and estimated (dotted line) breeding values averaged over each of the six years of selection for the benchmark (BEN), 40% pedigree error (PE-40) and weak connectedness (WCO) scenarios. Different colors represent different scenarios

We specifically focus on reporting the genetic gains for contrasting scenarios, as shown in Fig. 3 (BEN, PE-40 and WCO; left, center and right panel, respectively). After six years of selection based on EBVs, the means of the TBVs were 2.73, 2.55 and 2.10 genetic standard deviations (${\sigma }_{g}$) for BEN, PE-40 and WCO, respectively, while those of the EBVs were 2.73, 1.59 and 2.47 ${\sigma }_{g}$. The breeding scheme under the benchmark scenario was the one that achieved the highest genetic gain, as we expected. Changes in the TBVs in this scenario perfectly matched those of EBVs. In contrast, for the PE-40 and WCO scenarios the true breeding values were systematically under- and over-estimated, respectively, which resulted in differences in the genetic gains. As expected, the genetic gains for the PE-25 and SCO scenarios were between the values obtained for the extreme scenarios (PE-40 and WCO, respectively) and BEN.

### Pedigree errors

There was no bias in the estimation of breeding values with complete pedigree, as expected. In contrast, errors in the pedigree induced bias (Table 1). The PE-25 scenario generated a true bias close to − 0.13 ${\sigma }_{g}$. When errors in the pedigree increased to 40% per year (PE-40), the bias further increased although not proportionally.

**Table 1:** Comparison between estimates and true values of bias (${\widehat{\Delta }}_{p},$ expressed as ${\sigma }_{g}$) and dispersion (${\widehat{b}}_{w,p}$) for the benchmark scenario (BEN) and scenarios with pedigree errors (PE-25 and PE-40)

Estimator	Scenario	Estimate (SD)	True (SD)	^a^Correlation estimated—true
${\widehat{\Delta }}_{p}$	BEN	0.002 (0.035)	0.003 (0.047)	0.799
${\widehat{\Delta }}_{p}$	PE-25	0.044 (0.038)	− 0.130 (0.068)	0.663
${\widehat{\Delta }}_{p}$	PE-40	0.070 (0.052)	− 0.180 (0.070)	0.604
${\widehat{b}}_{w,p}$	BEN	1.006 (0.090)	0.990 (0.134)	0.708
${\widehat{b}}_{w,p}$	PE-25	0.960 (0.161)	0.894 (0.189)	0.665
${\widehat{b}}_{w,p}$	PE-40	0.980 (0.123)	1.054 (0.218)	0.722

^a^Pearson correlations across different years in partial data set and replicates; Figures represent averages and standard deviations (SD) across replicates

**Fig. 4:**
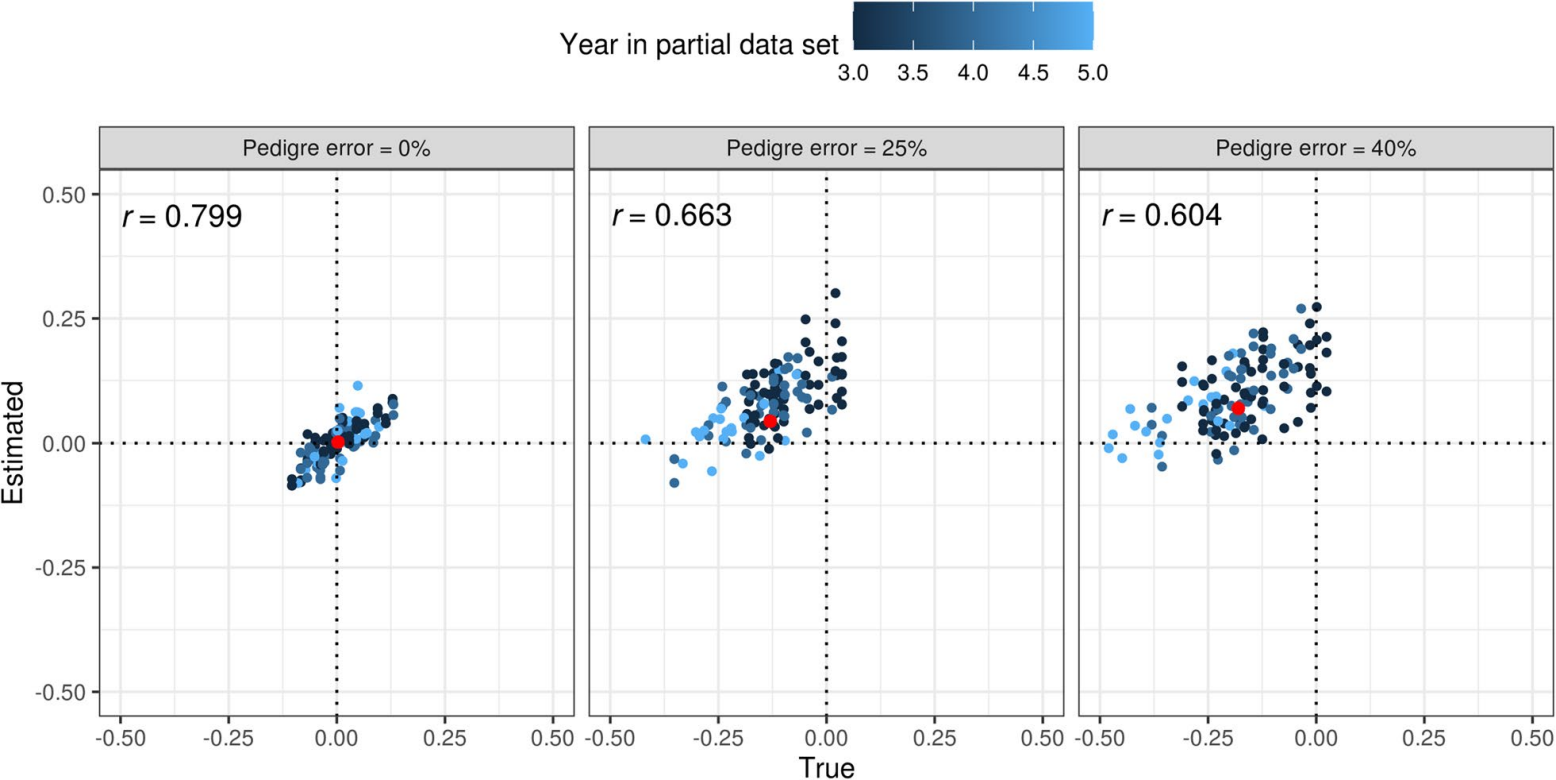
Changes in estimated versus true bias according to the % of total errors in the pedigree. In each plot, r denotes the Pearson correlation coefficient between true and estimated values. Different colors are used for different years in partial data set and red points indicate average biases from Table 1

Table 1 shows the estimates of bias (${\widehat{\Delta }}_{p}$) and dispersion $({\widehat{b}}_{w,p})$ in the different pedigree errors scenarios tested. For the BEN scenario, the true bias was correctly estimated in magnitude and direction. Conversely, the magnitude and direction of the bias induced by pedigree errors was on average not correctly estimated for the PE-25 and PE-40 scenarios. A strong positive correlation was observed between the true and estimated biases for the BEN scenario (corr(${\Delta }_{p}$, ${\widehat{\Delta }}_{p}$) ≈ 0.8; Fig. 4, left panel). This correlation decreased as errors in the pedigree increased, which also led to an increase in the magnitude of bias (Fig. 4, center and right panels).

Concerning the ${b}_{w,p}$, the true value was on average close to one, although it showed large deviations across replicates (based on its SD). In this case, the estimate performed reasonably well across the different scenarios, with a slight under and over-estimation for PE-25 and PE-40, respectively (Table 1). In general, there was a good agreement between the true and estimated values of this metric across years and replicates (corr(${b}_{w,p}$, ${\widehat{b}}_{w,p}$) =  ~ 0.7) for BEN and PE-40.

With respect to the estimates of accuracies and reliabilities ratios (${\widehat{\rho }}_{w,p}$ and $\widehat{{\rho }_{w,p}^{2}}$), they were on average closely aligned with their corresponding true values across all different scenarios (Table 2). In addition, we found good agreement between estimates and true values from different years and replicates, with correlations > 0.7 in both metrics, except for $\widehat{{\rho }_{w,p}^{2}}$ in the PE-40 scenario. A similar behavior was found between estimated and true values of the unselected reliability of $EB{V}_{p}$ metric for BEN, PE-25, and PE-40 scenarios (Table 2). It is noticeable that ${\widehat{rel}}_{p}$ is more accurately estimated (based on its estimated-true correlations) than ${\widehat{\rho }}_{w,p}$ or $\widehat{{\rho }_{w,p}^{2}}$.

**Table 2:** Comparison between estimates and true values of accuracies (${\widehat{\rho }}_{w,p}$), reliabilities ($\widehat{{\rho }_{p,w}^{2}}$) ratios, and unselected reliabilities (${\widehat{rel}}_{p}$) for the benchmark scenario (BEN) and the scenarios with pedigree errors (PE-25 and PE-40)

Estimator	Scenario	Estimate (SD)	True (SD)	^a^Correlation estimated—true
${\widehat{\rho }}_{w,p}$	BEN	0.468 (0.043)	0.444 (0.064)	0.773
${\widehat{\rho }}_{w,p}$	PE-25	0.464 (0.078)	0.419 (0.079)	0.750
${\widehat{\rho }}_{w,p}$	PE-40	0.497 (0.074)	0.515 (0.098)	0.692
$\widehat{{\rho }_{p,w}^{2}}$	BEN	0.219 (0.029)	0.210 (0.057)	0.705
$\widehat{{\rho }_{p,w}^{2}}$	PE-25	0.227 (0.046)	0.195 (0.067)	0.682
$\widehat{{\rho }_{p,w}^{2}}$	PE-40	0.256 (0.050)	0.294 (0.105)	0.570
${\widehat{rel}}_{p}$	BEN	0.527 (0.043)	0.536 (0.030)	0.905
${\widehat{rel}}_{p}$	PE-25	0.495 (0.067)	0.501 (0.063)	0.955
${\widehat{rel}}_{p}$	PE-40	0.479 (0.072)	0.513 (0.062)	0.912

^a^ Pearson correlations across different years in partial data set and replicates; Figures represent averages and standard deviations (SD) across replicates

### Connectedness

Comparisons between the estimated and true herd effects in the SCO and WCO scenarios revealed insights into the precision of these estimates relative to the true values set in the simulation (*H*_1_ = 2, *H*_2_ = 1, and *H*_3_ = 0). The trend towards overestimation became more pronounced in the WCO scenario, as was observed for the $\widehat{{H}_{1}}$ (3.41, 95%CI = 3.40 to 3.41) and $\widehat{{H}_{2}}$ (1.64, 95%CI = 1.63 to 1.65) mean herd effects. Conversely, this pattern was less pronounced in SCO, where $\widehat{{H}_{1}}$ and $\widehat{{H}_{2}}$ had a lower overestimation (2.51 and 1.25, 95%CI = 2.45 to 2.56 and 1.22 to 1.27, respectively). To maintain the same comparative baseline as in the true herd effects values, the means were adjusted so that the mean in $\widehat{{H}_{3}}$ was zero.

**Fig. 5:**
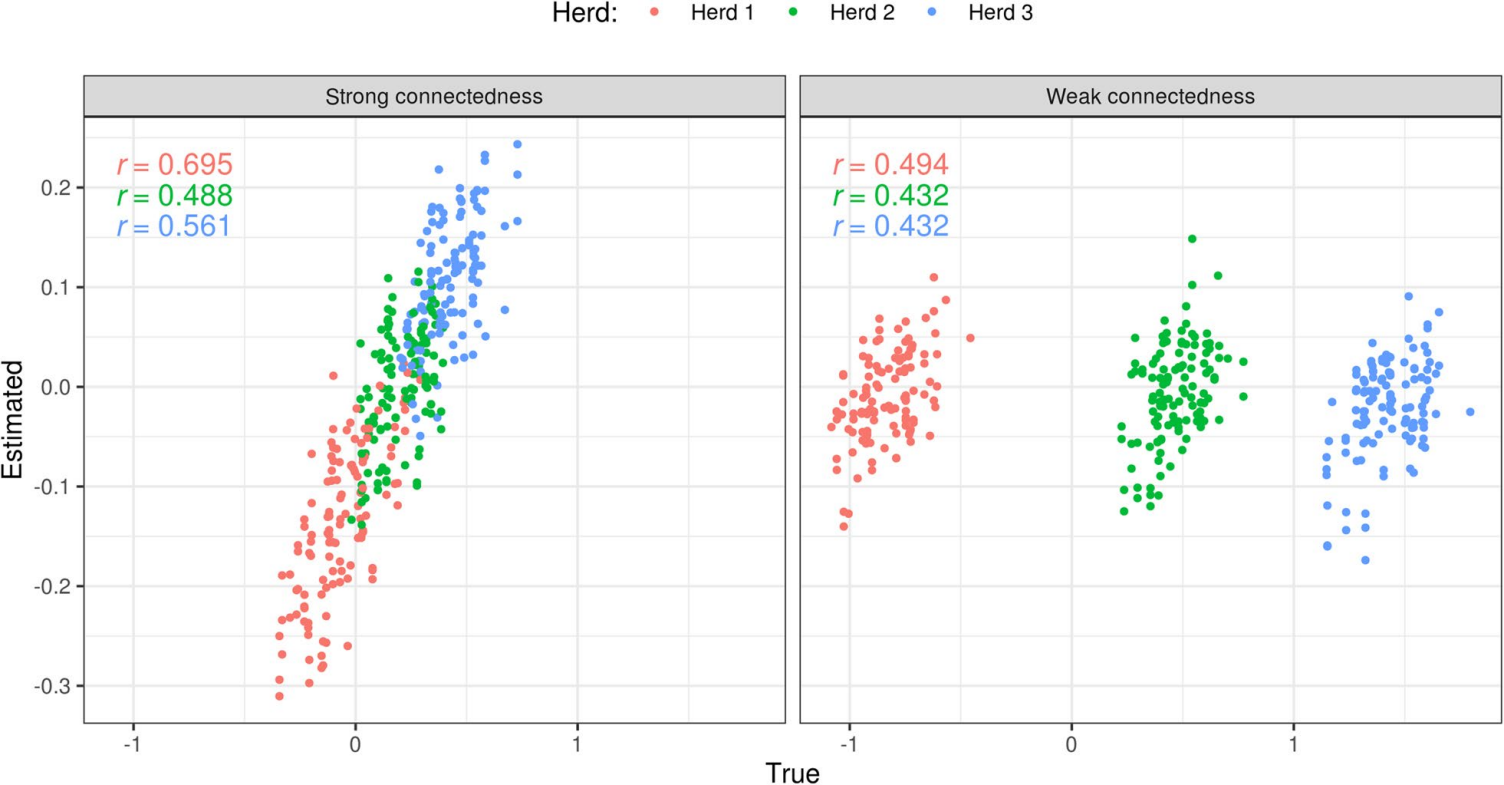
Changes in estimated versus true bias when different levels of connectedness were simulated. In each plot, r denotes the Pearson correlation coefficients between true and estimated values. Different colors are used for different herds

Differences in bias were observed between herds in the scenario with weak connectedness (WCO, Table 3). The magnitude of this bias is explained by both the herd effects and their different genetic levels. Animals in herds 1 and 3, the extreme ones, presented the strongest true biases for WCO (– 0.84 and 1.43 ${\sigma }_{g}$, respectively, Table 3). An under- and over-estimation of the TBVs of target bulls were caused by confounding: due to the lack of connections, the genetic evaluation model was not able to disentangle the genetic effect of bulls performing within the herd from the herd’s environmental effects. In general, the bias estimator (${\widehat{\Delta }}_{p}$) could not correctly estimate the magnitude and direction of this bias (Table 3 and Fig. 5). For example, in the herd 3 the true bias was close to 1.4 ${\sigma }_{g}$ but the corresponding estimate was close to zero.

**Table 3:** Comparison between estimates and true values of bias (${\widehat{\Delta }}_{p}$, expressed as ${\sigma }_{g}$) and dispersion (${\widehat{b}}_{w,p}$) in scenarios of weak (WCO) and strong (SCO) connectedness

Estimator	Scenario	Herd	Estimate (SD)	True (SD)	^a^Correlation estimated—true
${\widehat{\Delta }}_{p}$	WCO	1	− 0.009 (0.030)	− 0.836 (0.114)	0.494
${\widehat{\Delta }}_{p}$	WCO	2	− 0.008 (0.030)	0.468 (0.103)	0.432
${\widehat{\Delta }}_{p}$	WCO	3	0.013 (0.033)	1.423 (0.122)	0.432
${\widehat{\Delta }}_{p}$	SCO	1	− 0.085 (0.041)	− 0.070 (0.108)	0.695
${\widehat{\Delta }}_{p}$	SCO	2	0.000 (0.043)	0.202 (0.087)	0.488
${\widehat{\Delta }}_{p}$	SCO	3	0.079 (0.038)	0.422 (0.089)	0.561
${\widehat{b}}_{w,p}$	WCO	1	0.966 (0.189)	0.992 (0.269)	0.763
${\widehat{b}}_{w,p}$	WCO	2	1.026 (0.190)	0.937 (0.300)	0.786
${\widehat{b}}_{w,p}$	WCO	3	1.032 (0.219)	0.955 (0.238)	0.631
${\widehat{b}}_{w,p}$	SCO	1	1.088 (0.239)	0.975 (0.254)	0.798
${\widehat{b}}_{w,p}$	SCO	2	1.039 (0.198)	1.157 (0.268)	0.709
${\widehat{b}}_{w,p}$	SCO	3	1.043 (0.169)	1.088 (0.289)	0.791

^a^Pearson correlations across different years in partial data set and replicates; Figures represent averages and standard deviations (SD) across replicates

On the other hand, in the scenario with strong connectedness (SCO) the herd-year effects introduced in the genetic evaluation model were better able to capture real environmental differences due to herds. In this case, observed differences in EBVs between the bulls in the partial and whole datasets indicated the bias in the correct direction (corr(${\Delta }_{p}$, ${\widehat{\Delta }}_{p}$) > 0.49; Fig. 5 left panel), although the magnitude was underestimated for herds 2 and 3 (Table 3). In this scenario the magnitude of the true bias was lower than in the WCO scenario.

Regarding the dispersion of EBV, the true slopes did not differ markedly from one and no substantial difference was observed (based on its 99% confidence intervals; not shown) in the estimates (${\widehat{b}}_{w,p}$), neither between herds nor among levels of connectedness. All the dispersion estimates for WCO and SCO were close to one (Table 3), indicating that there was neither over- nor under-dispersion.

The ${\widehat{\rho }}_{w,p}$ and $\widehat{{\rho }_{w,p}^{2}}$ estimates were similar between weak and strong connectedness scenarios (Table 4) and showed good agreement with their true values, with correlations between estimates and true values across replicates and years greater than 0.5. Finally, regarding the ${rel}_{p}$, its estimates remained nearly constant across connectedness levels and herds, with values close to 0.50 (Table 4). This parameter was, as in pedigree-based scenarios, very well estimated (corr(${\widehat{rel}}_{p}$,$re{l}_{p}$) > 0.9).

**Table 4:** Comparison between estimates and true values of accuracies (${\widehat{\rho }}_{w,p}$), reliabilities ($\widehat{{\rho }_{p,w}^{2}}$) ratios, and unselected reliabilities (${\widehat{rel}}_{p}$) in scenarios of weak (WCO) and strong (SCO) connectedness

Estimator	Scenario	Herd	Estimate (SD)	True (SD)	^a^Correlation estimated—true
${\widehat{\rho }}_{w,p}$	WCO	1	0.456 (0.081)	0.444 (0.097)	0.747
${\widehat{\rho }}_{w,p}$	WCO	2	0.489 (0.058)	0.426 (0.129)	0.758
${\widehat{\rho }}_{w,p}$	WCO	3	0.448 (0.088)	0.424 (0.082)	0.697
${\widehat{\rho }}_{w,p}$	SCO	1	0.495 (0.089)	0.412 (0.100)	0.774
${\widehat{\rho }}_{w,p}$	SCO	2	0.485 (0.068)	0.512 (0.107)	0.717
${\widehat{\rho }}_{w,p}$	SCO	3	0.485 (0.077)	0.479 (0.107)	0.798
$\widehat{{\rho }_{p,w}^{2}}$	WCO	1	0.219 (0.047)	0.222 (0.085)	0.567
$\widehat{{\rho }_{p,w}^{2}}$	WCO	2	0.239 (0.033)	0.214 (0.109)	0.617
$\widehat{{\rho }_{p,w}^{2}}$	WCO	3	0.200 (0.050)	0.200 (0.077)	0.751
$\widehat{{\rho }_{p,w}^{2}}$	SCO	1	0.231 (0.043)	0.198 (0.080)	0.691
$\widehat{{\rho }_{p,w}^{2}}$	SCO	2	0.235 (0.043)	0.287 (0.104)	0.574
$\widehat{{\rho }_{p,w}^{2}}$	SCO	3	0.230 (0.045)	0.257 (0.109)	0.589
${\widehat{rel}}_{p}$	WCO	1	0.550 (0.064)	0.546 (0.052)	0.924
${\widehat{rel}}_{p}$	WCO	2	0.541 (0.077)	0.537 (0.063)	0.920
${\widehat{rel}}_{p}$	WCO	3	0.529 (0.071)	0.531 (0.056)	0.926
${\widehat{rel}}_{p}$	SCO	1	0.487 (0.091)	0.507 (0.064)	0.926
${\widehat{rel}}_{p}$	SCO	2	0.531 (0.066)	0.552 (0.065)	0.921
${\widehat{rel}}_{p}$	SCO	3	0.526 (0.083)	0.537 (0.074)	0.885

^a^Pearson correlations across different years in partial data set and replicates; Figures represent averages and standard deviations (SD) across replicates

## Discussion

In this study we evaluated the performance of the LR method [5] as a validation tool for genetic evaluation programs through a simulation experiment. Specifically, our focus was on complex scenarios typically found in beef cattle populations. The scenarios involved different levels of pedigree errors and genetic connectedness, two challenges frequently encountered in these genetic evaluation programs.

The LR method assumes that the genetic evaluation model meets the BLUP theory assumptions, in general, and the existence of a complete and accurate pedigree and genetic connections among herds, in particular. Our BEN scenario fulfilled these assumptions and showed very good agreement between the true values of the LR metrics and their estimates. This aligns with the results obtained by Macedo et al. [6], who found that, as long as the model is correct, the pedigree complete and the population well-connected, the LR method provides robust estimates of all of its metrics. However, both errors in the pedigree and severe lack of connectedness induced a strong bias in the genetic evaluations that, we observed, the corresponding LR statistic ${\widehat{\Delta }}_{p}$ was not able to accurately capture.

Pedigree errors involve the introduction of spurious relationships between the animals that mask the true relationships. This can lead to biased breeding values and genetic parameter estimates [14, 15]. One common effect of pedigree errors is that they tend to shrink all the estimated breeding values toward the population mean. This effect is even more severe for the parents that are in the extremes of the distribution (i.e. highest and lowest breeding values; [28]). When the percentage of errors in the pedigree is high and many parents have their EBVs biased, the resulting offspring will tend to have less extreme EBVs. Consequently, the selection of superior animals will be inaccurate, often favoring the selection of younger bulls [25], and the estimated genetic trends will poorly estimate the actual genetic gain (as discussed below).

In scenarios where pedigree errors were introduced at rates of 25% and 40% (PE-25 and PE-40), we observed that the LR method detected biases. However, it did not estimate these biases accurately. The underlying factors contributing to the LR method's tendency to estimate bias in the opposite direction remain unclear. It's crucial to highlight that while the LR method detects the presence of bias, it doesn't offer definitive insight into the specific causes of these biases. In a nutshell, in scenarios involving pedigree errors, the LR method sheds light on potential bias problems in the genetic evaluation, although it does not provide a complete picture of why biases occur.

Another challenge in beef cattle genetic evaluation is dealing with weak connectedness, which refers to the situation where there are few closely related animals in the pedigree across management units [12, 13]. This is particularly important in beef cattle breeds compared to dairy cattle because beef cattle populations use less AI bulls. Weak connectedness should not bias the estimates of breeding values, as long as the animals are randomly distributed in the population with respect to their true breeding values [13]. However, if this assumption does not hold, bias can occur. This is particularly true in situations where one country produces genetic improvement and another heavily imports this genetic material. Examples include breeds such as Angus or Holstein cattle, or improved pig lines. In these cases, the information on which the selection was based may not be available. As a result, genetic and environmental effects can become confounded, especially in scenarios of weak connectedness. To mimic this lack of connectedness in our study, we violated the assumption and simulated two scenarios where genetic and environmental effects became strongly (WCO) or weakly (SCO) confounded within three distinct herds, both of which led to bias in genetic evaluations. In the scenario where the lack of connectedness was stronger (WCO), the LR method failed to detect the true bias generated. Instead, when the connectedness was higher (SCO), the LR statistic was able to detect bias in one of the three herds, though it remained undetected in the others.

As discussed earlier, the biases found in genetic evaluations have implications when estimating genetic gains by means of genetic trends. To avoid over- or under-estimation of genetic gain, we basically need an *unbiasedness* condition; that is, the means of EBVs should be the same as the means of TBVs for all selection candidates. This ensures fair comparisons across old and young animals and is particularly important in breeding programs with complex age structures [4]. Instead, we expect over- or under-estimation of genetic gain when bias is positive or negative, respectively. We observed this in our study when contrasting the benchmark scenarios with those involving errors in the pedigree and lack of connectedness. These scenarios led to less genetic gain than expected (Fig. 3).

Reliable data collection procedures and maintenance of accurate and complete pedigree records are required to minimize the impact of pedigree errors and connectedness problems on genetic evaluations. The above may also involve validating parentage information through DNA testing, for example. In the genomic era, molecular markers can provide information on the genetic relationships among animals that is not captured by the pedigree. Also, they may help refining relationships between animals from different management units [29, 30]. Although our study did not address the LR metrics using estimates of genomic breeding values (GEBVs), it is expected that genomic data would have improved connections between management units, for example in the WCO scenario, provided that a significant proportion (or all) of the animals were genotyped. This would lead to a less biased genetic evaluation model, better capturing real environmental differences due to the herds, as seen in the SCO scenario.

Another approach could be to use more sophisticated statistical models that can account for the uncertainty and sparseness of the pedigree data (see Masuda et al. [31] for a comprehensive review of models for missing pedigree). For example, the inclusion of genetic groups or metafounders (MF) in genetic evaluation models has been proposed to address biases associated with large missing pedigree [32, 33]. Macedo et al. [27] and Kluska et al. [34], applied unknown parent groups (UPG) and MF to model missing pedigree data within dairy sheep and composite cattle populations, respectively. The findings of these works, which used the LR method to estimate ${\widehat{\Delta }}_{p}$, revealed that MF significantly reduces bias and, when incorporated into genomic models, produces less biased genomic predictions. Interestingly, Macedo et al. [35] suggested that removing old data in the pedigree, regardless of including UPG or MF in the genetic evaluation model, is an efficient and practical strategy to alleviate this kind of bias.

In summary, mitigating the bias resulting from these common issues in beef cattle genetic evaluations, requires meticulous record collection and the implementation of appropriate statistical models. Our results indicate that the LR method's metric ${\widehat{\Delta }}_{p}$ may not perform adequately under certain conditions of pedigree errors and weak connectedness. It would be prudent for studies using the LR method to report detailed information about the extent of missing pedigree and measures of connectedness, as these factors significantly impact the assumptions underlying most animal genetic evaluation models. Fitting UPG into the model may improve the estimator’s performance by addressing some of these errors (missing pedigree). However, accurate estimation of UPGs needs strong connectedness [36], which may not always be feasible. While MF can help mitigate these issues, it is not clear whether it definitively resolves them or consequently improves the performance of the LR method's metric ${\widehat{\Delta }}_{p}$; further investigation is required.

In our examination of the LR dispersion metric, we consistently observed that even in scenarios involving pedigree errors and connectedness problems, the true dispersion values ranged between 0.9 and 1.05, indicating an absence of inflation or deflation, respectively, in EBVs. Moreover, a good agreement was observed between the true dispersion values and those estimated by the LR method for BEN and PE-40. Dispersion values close to one indicated that the EBVs of the focal individuals were consistently expressed on a uniform scale, irrespective of whether their progeny had recorded data in the genetic evaluation, across all the scenarios under consideration. While inflation doesn't affect the ranking of animals within the same generation, it becomes relevant when selecting across generations, potentially favoring unproven young candidates over proven ones [37].

With respect to the estimates of ratios of accuracies and reliabilities, they all behaved quite stable within each evaluated scenario. In our study, the source of information that was added to improve the accuracies and reliabilities of the EBVs of the sires (our focal group) when moving from the partial to the whole data set was the phenotypic records of their offspring (own phenotypic records were already present in the partial data set). Especially, the LR metric ${\widehat{\rho }}_{w,p}$ reflected this increase and showed an acceptable agreement with their true counterparts even in the scenarios with errors in the pedigree and weak connectedness. These results agree with those reported by Macedo et al. [6], who pointed out that this may be due to the fact that the ratio of accuracies, which is based on correlation, is invariant to changes in the mean or scale of the data used to estimate it. It is important to emphasize that although both estimators of ratios of accuracies and reliabilities are expected to be equivalent, meaning $E\left({\widehat{\rho }}_{w,p}\right)=\sqrt{E\left(\widehat{{\rho }_{w,p}^{2}}\right)}$, individual realizations of these estimators (${\widehat{\rho }}_{w,p}$ and $\sqrt{\widehat{{\rho }_{w,p}^{2}}}$) are not necessarily equal [5]. Additionally, ${\widehat{\rho }}_{w,p}$ is not influenced by simple forms of overdispersion (${\widehat{b}}_{p}$< 1), unlike $\widehat{{\rho }_{w,p}^{2}}$ which requires the evaluation model to be unbiased (${\widehat{b}}_{p}$= 1) to achieve its expected value. Therefore, if ${\widehat{b}}_{p}$≠1, the estimators are likely to differ [6].

Finally, it is relevant to address the unselected reliabilities estimator derived from the LR method, which resembles the theoretical accuracies derived from the inverse of the MME [27]. This metric has been theoretically addressed [6, 27] but is seldomly explored practically. Its computation requires an estimate of the additive genetic variances of both focal individuals (${\sigma }_{a*}^{2}$) and the base population (${\sigma }_{a}^{2}$). In our study, we used the true variances to obtain ${\widehat{rel}}_{p}$, which contributed both to the stability of these estimates across different scenarios and to a better agreement between the true and estimated metrics.

## Conclusions

The LR method is not able to reveal the bias in estimated breeding values induced by severe cases of incomplete pedigrees or lack of connectedness. However, even under these extreme scenarios, the method is a useful tool for estimating and evaluating dispersion of EBV and increase of accuracies and reliabilities.

## Supplementary Information

Supplementary Material 1. Genetic connectedness measures used in the simulated scenarios (“Lack of connectedness”).

Supplementary Material 2. Figure S1 Connectedness estimates across contemporary groups based on average scaled PEVD for both connectedness scenarios. We provide a detailed view of the degree of connectedness based on pairwise prediction error variance of difference (PEVD) values between CGs in two of the simulated scenarios: strong connectedness (SCO; left panel) and weak connectedness (WCO; right panel), from selection year 1 to 6. Note: the PEVD estimates are averages across 20 replicates, with standard deviations ranging from 0.000 and 0.003. Figure S2 Differences in PEVD values between connectedness scenarios. Here we show the difference in the pairwise prediction error variance of difference (PEVD) between the two connectedness scenarios: weak connectedness (WCO) minus strong connectedness (SCO). Red dashed lines delineate the cases where CG comparisons involve pairs of herds across years. Figure S3 Connectedness estimates across contemporary groups based on average GDV* for both connectedness scenarios. We show the degree of connectedness based on the genetic drift variance (GDV*) values between contemporary groups in two of the simulated scenarios: strong connectedness (SCO; left panel) and weak connectedness (WCO; right panel), from selection year 1 to 6. Note: the GDV* estimates are averages across 20 replicates, with standard deviations ranging from 0.00 to 0.03. Figure S4 Common sires across contemporary groups for both connectedness scenarios. Here we plot the number of common sires between the different contemporary groups (herd-year) according to the simulated strategy to achieve the two levels of connectedness: strong (right panel) and weak (left panel). Values (colours) represent averages across the 20 replicates. Red dashed lines delineate pairs of herds across years. Figure S5 Plots of the first two principal components (PC) based on the genomic relationship matrix for both connectedness scenarios. We show the effects of simulated connectedness levels across herds on genomic relationship in selection years 1 and 6 (left and right panels, respectively). After quality control (minor allele frequency > 0.01), the total number of SNPs retained for each PCA ranged from 83,168 to 89,500, depending on the population and the scenario analysed: 4300 animals in both the year one and six of selection for each scenario (WCO and SCO; top and bottom panels, respectively). The PCA was applied to the genomic relationship matrix calculated following VanRaden [41]. Colours within each panel indicate the herds (1 to 3) and sex of animals (cows and bulls). Note: the example was taken from the data of the first replicate

## Data Availability

The datasets used and/or analysed during the current study are available from the corresponding author on reasonable request.

## Appendix

### Averaging out estimates of the LR metrics

In the main text of our article, we explain that for each LR metric within a scenario and a replicate we obtained six different estimates. These arouse from comparing “partial” breeding values from bulls born either in year ${n}_{p} ({n}_{p}=\left\{3, 4, 5\right\})$ with the corresponding “whole” breeding values computed on years ${n}_{w}={n}_{p}+1 ({n}_{w}=\left\{4, 5, 6\right\})$. To average out these estimates, we followed the procedure described by Macedo et al. [27]. Here we describe this procedure in more detail.

Briefly, the procedure involves two steps. In the first step, a two-way ANOVA with no interaction model is fitted to the six LR metric estimates. The two factors are related to whether the estimate was obtained with partial breeding values from year ${n}_{p}$ and whole breeding values from year ${n}_{w}$ or not, and thus involve three levels each. To be more explicit, consider the values obtained for the bias in one of the replicates and the corresponding linear model in matrix notation:

$$\underbrace {{\left[ {\begin{array}{*{20}c} { - 0.02384} \\ { - 0.02057} \\ { - 0.02193} \\ {0.01461} \\ {0.02103} \\ {0.07263} \\ \end{array} } \right]}}_{{\varvec{y}}} = \underbrace {{\left[ {\begin{array}{*{20}c} 1 & 1 & 0 & 0 & 1 & 0 & 0 \\ 1 & 1 & 0 & 0 & 0 & 1 & 0 \\ 1 & 1 & 0 & 0 & 0 & 0 & 1 \\ 1 & 0 & 1 & 0 & 0 & 1 & 0 \\ 1 & 0 & 1 & 0 & 0 & 0 & 1 \\ 1 & 0 & 0 & 1 & 0 & 0 & 1 \\ \end{array} } \right]}}_{{\mathbf{X}}} = \underbrace {{\left[ {\begin{array}{*{20}c} \mu \\ {\alpha_{p1} } \\ {\alpha_{p2} } \\ {\alpha_{p3} } \\ {\alpha_{w1} } \\ {\alpha_{w2} } \\ {\alpha_{w3} } \\ \end{array} } \right]}}_{{{\varvec{\upalpha}}}} + \underbrace {{ {\left[ {\begin{array}{*{20}c} {\varepsilon_{1} } \\ {\varepsilon_{2} } \\ {\varepsilon_{3} } \\ {\varepsilon_{4} } \\ {\varepsilon_{5} } \\ {\varepsilon_{6} } \\ \end{array} } \right]}}}_{{{\varvec{\upvarepsilon}}}}$$

This is, of course, an overparameterized model, and consequently, least-square solutions are not unique. However, by dropping columns two and five of the **X** matrix above an equivalent model is obtained:

$$\underbrace {{\left[ {\begin{array}{*{20}c} { - 0.02384} \\ { - 0.02057} \\ { - 0.02193} \\ {0.01461} \\ {0.02103} \\ {0.07263} \\ \end{array} } \right]}}_{{\varvec{y}}} = \underbrace {{\left[ {\begin{array}{*{20}c} 1 & 0 & 0 & 0 & 0 \\ 1 & 0 & 0 & 1 & 0 \\ 1 & 0 & 0 & 0 & 1 \\ 1 & 1 & 0 & 1 & 0 \\ 1 & 1 & 0 & 0 & 1 \\ 1 & 0 & 1 & 0 & 1 \\ \end{array} } \right]}}_{{{\mathbf{X}^{*}}}} = \underbrace {{\left[ {\begin{array}{*{20}c} \mu \\ {\alpha_{p2}^{{*}} } \\ {\alpha_{p3}^{{*}} } \\ {\alpha_{w2}^{{*}} } \\ {\alpha_{w3}^{{*}} } \\ \end{array} } \right]}}_{{{\mathbf{\alpha * }}}} + \underbrace {{\left[ {\begin{array}{*{20}c} {\varepsilon_{1}^{{*}} } \\ {\varepsilon_{2}^{{*}} } \\ {\varepsilon_{3}^{{*}} } \\ {\varepsilon_{4}^{{*}} } \\ {\varepsilon_{5}^{{*}} } \\ {\varepsilon_{6}^{{*}} } \\ \end{array} } \right]}}_{{{\mathbf{\varepsilon * }}}},$$

where the following parametric equivalence are established:

$${\alpha }_{p2}^{{*}}={\alpha }_{p1}- {\alpha }_{p2}, {\alpha }_{p3}^{{*}}={\alpha }_{p1}- {\alpha }_{p3}, {\alpha }_{w2}^{{*}}={\alpha }_{w1}- {\alpha }_{w2} \, and \, {\alpha }_{w2}^{{*}}={\alpha }_{w1}- {\alpha }_{w3}.$$

In the second step of the procedure, an estimable function that yields a unique estimate “as if the design was balanced” [27] is calculated. For the reparametrized model, the coefficients of this function are:

$${\mathbf{c}}^{T}=\left[\begin{array}{ccccc}1& \frac{1}{2}& \frac{1}{2}& \frac{1}{2}& \frac{1}{2}\end{array}\right]$$

Thus, in our example and for this scenario and replicate, the estimate of the bias is:

$${\mathbf{c}}^{T}\widehat{{{\varvec{\upalpha}}}^{*}}=\left[\begin{array}{ccccc}1& \frac{1}{2}& \frac{1}{2}& \frac{1}{2}& \frac{1}{2}\end{array}\right]\left[\begin{array}{c} \widehat{\mu } \\ \widehat{{\alpha }_{p2}^{*} }\\ \widehat{{\alpha }_{p3}^{*}} \\ \widehat{{\alpha }_{w2}^{*}} \\ \widehat{{\alpha }_{w3}^{*}}\end{array}\right]=0.00446.$$

For each LR metric within the replicate, the linear model was fitted using the ‘lm’ function in R and the estimate was produced resorting to the ‘estimate’ function from the R package ‘gmodels’ [42].
