# Supplementary material for: On the ability of the LR method to detect bias when there is pedigree misspecification and lack of connectedness
Source: Genet Sel Evol. 2024 Nov 21;56:74. doi: 10.1186/s12711-024-00943-1 (PMC11583403; doi:10.1186/s12711-024-00943-1)
Supplement: Supplementary file 1 — Supplementary Material 1. Genetic connectedness measures used in the simulated scenarios (“Lack of connectedness”). [file 12711_2024_943_MOESM1_ESM.docx]

Genetic connectedness measures used in the simulated scenarios (“Lack of connectedness”)

The strategy we used to simulate the strong connectedness (SCO) and weak connectedness (WCO) scenarios may seem unintuitive. A key concern with this strategy is whether strong and weak connections between herds were indeed achieved. We address this issue here. Specifically, we define the concept of genetic connectedness and describe in detail the measures we used to ensure that our scenarios were truly contrasting with respect to this property.

Genetic connectedness is a measure of the extent to which estimated breeding values (EBV) can be fairly compared across contemporary groups (CGs). It is typically assessed using the inverse of the coefficient matrix derived from Henderson’s mixed model equations (MME; [11]). Kennedy and Trus [12] introduced a measure of genetic connectedness based on the average prediction error variance of the difference (PEVD) in predicted breeding values between pairs of individuals from different contemporary groups. They also proposed a related measure, termed ‘genetic drift variance’ (GDV), as an alternative to PEVD, and reported a high correlation (>0.9) between the two measures. PEVD-derived statistics are often considered the most accurate for estimating genetic connectedness [12]. However, their interpretation is challenging due to the absence of a benchmark and the fact that they operate within a narrow range, making differences difficult to interpret. Additionally, the PEVD-based connectedness metric is unable to identify completely disconnected CGs. In contrast, GDV can take values over a broader range that can be easily mapped to the (0, 1) interval, allowing for a clearer distinction between connected and disconnected CGs.

To be more precise, we first need to define the prediction error variance (PEV) of breeding values. Consider the standard univariate Animal model typically used in genetic evaluation:

$$\mathbf{y=Xh+Za+e}$$

where $\mathbf{y}$ is the vector of records, $\mathbf{X}$ and $\mathbf{Z}$ are incidence matrices for herd-year ($\mathbf{h}$) and animals’ breeding values ($\mathbf{a}$), respectively, and $\mathbf{e}$ is the vector of errors. The elements of $\mathbf{a}$ and $\mathbf{e}$ are identifiable given that they have known covariance matrices, denoted $Var(\mathbf{a})=\mathbf{A}\sigma_{a}^{2}$ and $Var(\mathbf{e})=\mathbf{I}\sigma_{e}^{2}$, respectively. $\mathbf{A}$ is the pedigree-based relationship matrix.

We can express the EBV of the animals in $\mathbf{a}$ as:

$$\hat{\mathbf{a}}\mathbf{=}\boldsymbol{C}^{\boldsymbol{-1}}\mathbf{Z'My}$$

where $\mathbf{C}^{\boldsymbol{-}\boldsymbol{1}}\mathbf{=}{\mathbf{(}\mathbf{Z}\mathbf{'}\mathbf{MZ}\mathbf{+}{\lambda\mathbf{A}}^{\mathbf{-}\mathbf{1}})}^{\mathbf{-}\mathbf{1}}$ is the inverse of the coefficient matrix ($\mathbf{C}$), $\mathbf{M}\mathbf{=}\mathbf{I}\mathbf{-}\mathbf{X}{\mathbf{(}\mathbf{X}\mathbf{'}\mathbf{X}\mathbf{)}}^{\boldsymbol{-}}\mathbf{X}\mathbf{'}$ is the absorption matrix for $\mathbf{h}$, and $\lambda=\frac{\sigma_{e}^{2}}{\sigma_{a}^{2}}$. The four blocks of $\mathbf{C}$ are given by

$$\mathbf{C=}\left[ \begin{matrix} \mathbf{C}_{\mathrm{hh}} & \mathbf{C}_{\mathrm{ha}} \\ \mathbf{C}_{\mathrm{ah}} & \mathbf{C}_{\mathrm{aa}} \end{matrix} \right]\boldsymbol{=}\left[ \begin{matrix} \mathbf{X'X} & \mathbf{X'Z} \\ \mathbf{Z'X} & \mathbf{Z}^{\mathbf{'}}\mathbf{Z+}{\lambda\mathbf{A}}^{\mathbf{-1}} \end{matrix} \right]$$

and its inverse:

$$\mathbf{C}^{\boldsymbol{-1}}\mathbf{=}\left[ \begin{matrix} \mathbf{C}^{\mathrm{hh}} & \mathbf{C}^{\mathrm{ha}} \\ \mathbf{C}^{\mathrm{ah}} & \mathbf{C}^{\mathrm{aa}} \end{matrix} \right]\boldsymbol{=}\left[ \begin{matrix} \mathbf{X'X} & \mathbf{X'Z} \\ \mathbf{Z'X} & \mathbf{Z}^{\mathbf{'}}\mathbf{Z+}{\lambda\mathbf{A}}^{\mathbf{-1}} \end{matrix} \right]^{\boldsymbol{-1}}\boldsymbol{=}\left[ \begin{matrix} Var(\hat{\mathbf{h}}) & cov(\hat{\mathbf{h}},\left( \hat{\mathbf{a}}\mathbf{-a} \right)\mathbf{'}\boldsymbol{)} \\ cov(\left( \hat{\mathbf{a}}\mathbf{-a} \right),\hat{\mathbf{h}}\mathbf{'}\boldsymbol{)} & \mathrm{Var}\left( \hat{\mathbf{a}}\mathbf{-a} \right) \end{matrix} \right]$$

Letting $\hat{a}_{i}\mathbf{-}a_{i}$ be the prediction error of EBV for the *i*th individual ($\hat{a}_{i}$), then its variance (PEV*_i_*) is as follows [11]:

$$\mathrm{PEV}_{i}\mathbf{=}\mathrm{Var}\left( \hat{a}_{i}\mathbf{-}a_{i} \right)\boldsymbol{=}C_{ii}^{\mathrm{aa}}\sigma_{e}^{2}$$

where $C_{ii}^{\mathrm{aa}}$ represents the diagonal element of the $\mathbf{C}^{\mathrm{aa}}$ block referring to the *i*th animal.

*Prediction error variance of difference (PEVD)*

The pairwise PEVD of the difference between the EBVs of the *i*th and *j*th animals from different herds or CGs is given by:

$$\mathrm{PEVD}_{ij}\mathbf{=}\left[ \mathrm{PEV}_{i}+\mathrm{PEV}_{j}-2\mathrm{PEC}_{ij} \right]$$

$$=\left( C_{ii}^{\mathrm{aa}}-C_{ji}^{\mathrm{aa}}-C_{ij}^{\mathrm{aa}}+C_{jj}^{\mathrm{aa}} \right)\sigma_{e}^{2}$$

$$=\left( C_{ii}^{\mathrm{aa}}+C_{jj}^{\mathrm{aa}}-2C_{ij}^{\mathrm{aa}} \right)\sigma_{e}^{2}$$

where $C_{ii}^{\mathrm{aa}}$ and $C_{jj}^{\mathrm{aa}}$ represents the diagonal elements of the $\mathbf{C}^{\mathrm{aa}}$ block referring to the *i*th and *j*th animals, respectively, and $C_{ji}^{\mathrm{aa}}$ and $C_{ij}^{\mathrm{aa}}$ are the off-diagonal elements of $\mathbf{C}^{\mathrm{aa}}$ which in turn measure the covariance between the difference of EBVs and is denoted by PEC.

The principle behind using PEVD as a measure of connectedness is based on the fact that accurately estimated breeding values have smaller PEV and genetically related individuals across different CG exhibit positive PEC. Therefore, smaller PEVD values indicate stronger connections and more accurate comparisons of breeding values across CGs. To express PEVD free from units of measurement, it may be scaled by the additive genetic variance, as suggested by Kuehn et al. [38]. Finally, to summarize pairwise scaled PEVD between individuals at the CG level, these can be averaged as suggested by Yu et al. [39]: $\mathrm{PEVD}_{kl}=\frac{1}{n_{k}\times n_{l}}\sum\mathrm{PEVD}_{kl}$, where $n_{k}$ and $n_{l}$ represent the total number of records in CGs *k* and *l*, respectively, and $\sum\mathrm{PEVD}_{kl}$ denotes the sum of all pairwise differences between the two CGs.

*Genetic drift variance (GDV)*

Another measure of connectedness proposed by Kennedy and Trus [12] is the genetic drift variance (GDV), which is less computationally intensive than PEVD. This measure is based on averaging the genetic relationships between and within CGs. Let $\mathbf{B}\mathbf{=}\mathbf{X}\mathbf{'}\mathbf{ZAZ}\mathbf{'}\mathbf{X}$, then, the average genetic relationships among individuals from the same or different CGs can be obtained by dividing each diagonal element of $\mathbf{B}$ by the square of the number of records in each CG, and the off-diagonal elements by the product of the number of records in the CGs involved. The resulting matrix is a square matrix of order equal to the number of CGs and is denoted by $\bar{\mathbf{A}}$.

According to Kennedy and Trus [12], the entries of the $\bar{\mathbf{A}}$ matrix can be interpreted as the genetic components of the (co)variance of genetic drift between CGs. Then, the GDV can be computed as:

$$\mathrm{GDV}_{ij}\mathbf{=}\left[ \bar{A}_{ii}+\bar{A}_{jj}-2\bar{A}_{ij} \right]$$

where GDV*_ij_* is the degree of connectedness between the *i*th and *j*th CG, $\bar{A}_{ii}$ and $\bar{A}_{jj}$ are the corresponding diagonal entries of $\bar{\mathbf{A}}$ (genetic drift variance for *i*th and *j*th CG, respectively), and $\bar{A}_{ij}$ is the corresponding off-diagonal entry of $\bar{\mathbf{A}}$ (genetic drift covariance between *i*th and *j*th CG). Smaller values indicate higher degrees of connectedness [12]. The values of GDV between CGs can easily be mapped to the (0, 1) interval by applying the transformation proposed by Magaña-Valencia et al. [40]:

$${GDV}_{ij}^{*}\mathbf{=}1-\left[ (\bar{A}_{ii}+\bar{A}_{jj}-2\bar{A}_{ij})/(\bar{A}_{ii}+\bar{A}_{jj}) \right]$$

Now, ${GDV}_{ij}^{*}$ values close to one indicate strong connectedness, whereas values close to zero indicate disconnected CG [40].

*Genetic connectedness in the simulated scenarios*

To assess the level of connectedness achieved in our simulated scenarios (SCO and WCO), we computed these two measures—the averaged scaled PEVD and GDV* —for each of the generated data sets and replicates and summarized the results graphically (Fig. S1 to Fig. S3). The figures clearly show that we attained the level of connectedness that was asserted for each scenario. Since the CGs were defined by the “herd-year” combination, it is possible to assess connectedness within herds across years, or vice versa.

Overall, PEVD values between CGs are lower in the SCO scenario compared to WCO, indicating stronger connectedness in the former. In the WCO scenario, the higher PEVD values (represented by red tones in Fig. S1) indicate lower connectedness and potentially greater uncertainty in comparisons between CGs. The differences in PEVD values for CGs between scenarios (WCO minus SCO) are more clearly visualized in Fig. S2. In both scenarios, certain CGs show relatively lower PEVD values (e.g., CGs involved in years 2 to 4). This may be due to the replacement strategy design, which led to a higher frequency of shared sires during those years (Fig. S4). Within herds and across the six years of selection, the SCO scenario also shows lower PEVD values compared to WCO. However, these differences are smaller compared to those involving "between-herd" comparisons over the years (Fig. S2, red dashed lines). The latter also shows more pronounced differences towards the final years of selection (year six), which is consistent with the breeding strategy design in both scenarios (i.e., greater genetic distance).

On the other hand, the GDV* measures the degree of connectedness for WCO and SCO, based on the average relationships between and within CGs (Fig. S3). In SCO, an increase in average relationships between herds is observed over the years, indicating greater connectedness among CGs. In contrast, these relationships increase only within herds and across years for WCO. A key advantage of GDV* is its ability to detect completely disconnected herds, as it was evidenced for WCO, where all GDV* values for CGs involving comparisons between herds were zero. In SCO, the highest GDV* values (>0.9) occurred between CGs from different herds in year 6, while in year 1, these values between herds were the lowest (weaker connectedness). Within herd and across years, connectedness increases between contiguous years due to a higher frequency of common sires (Fig. S4), but decreases as the years progress, reflecting more distant connections. This trend is also observed between herds for more distant years, but in a more pronounced manner.

*Genomic distance in the simulated scenarios*

In addition, we performed a principal component analysis (PCA) using genomic data from the simulated populations to visualize the genomic relationships attained among the animals within the simulated herds after the simulation was completed (Fig. S5). The plots demonstrate that genomic differentiation responded to the design of each scenario. Importantly, while the PCA does not measure the level of connectedness, it illustrates its effects on the genomic distance achieved. The PCA was computed for a single replicate to illustrate this effect.
