# Supplementary material for: On the ability of the LR method to detect bias when there is pedigree misspecification and lack of connectedness
Source: Genet Sel Evol. 2024 Nov 21;56:74. doi: 10.1186/s12711-024-00943-1 (PMC11583403; doi:10.1186/s12711-024-00943-1)
Supplement: Supplementary file 2 — Supplementary Material 2. Figure S1 Connectedness estimates across contemporary groups based on average scaled PEVD for both connectedness scenarios. We provide a detailed view of the degree of connectedness based on pairwise prediction error variance of difference (PEVD) values between CGs in two of the simulated scenarios: strong connectedness (SCO; left panel) and weak connectedness (WCO; right panel), from selection year 1 to 6. Note: the PEVD estimates are averages across 20 replicates, with standard deviations ranging from 0.000 and 0.003. Figure S2 Differences in PEVD values between connectedness scenarios. Here we show the difference in the pairwise prediction error variance of difference (PEVD) between the two connectedness scenarios: weak connectedness (WCO) minus strong connectedness (SCO). Red dashed lines delineate the cases where CG comparisons involve pairs of herds across years. Figure S3 Connectedness estimates across contemporary groups based on average GDV* for both connectedness scenarios. We show the degree of connectedness based on the genetic drift variance (GDV*) values between contemporary groups in two of the simulated scenarios: strong connectedness (SCO; left panel) and weak connectedness (WCO; right panel), from selection year 1 to 6. Note: the GDV* estimates are averages across 20 replicates, with standard deviations ranging from 0.00 to 0.03. Figure S4 Common sires across contemporary groups for both connectedness scenarios. Here we plot the number of common sires between the different contemporary groups (herd-year) according to the simulated strategy to achieve the two levels of connectedness: strong (right panel) and weak (left panel). Values (colours) represent averages across the 20 replicates. Red dashed lines delineate pairs of herds across years. Figure S5 Plots of the first two principal components (PC) based on the genomic relationship matrix for both connectedness scenarios. We show the effects of simulated connectedness lev [file 12711_2024_943_MOESM2_ESM.docx]

**Figure S1 Connectedness estimates across contemporary groups based on average scaled PEVD for both connectedness scenarios.**


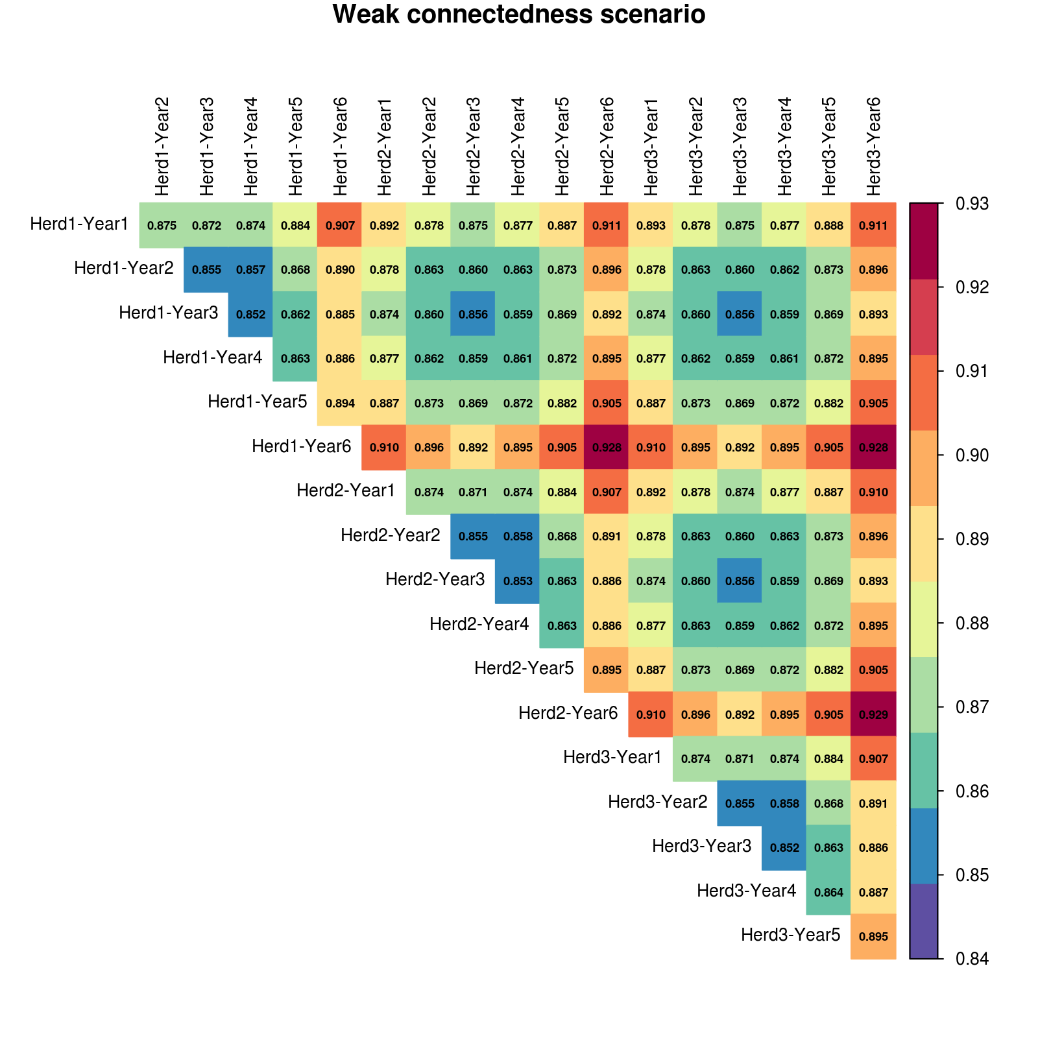

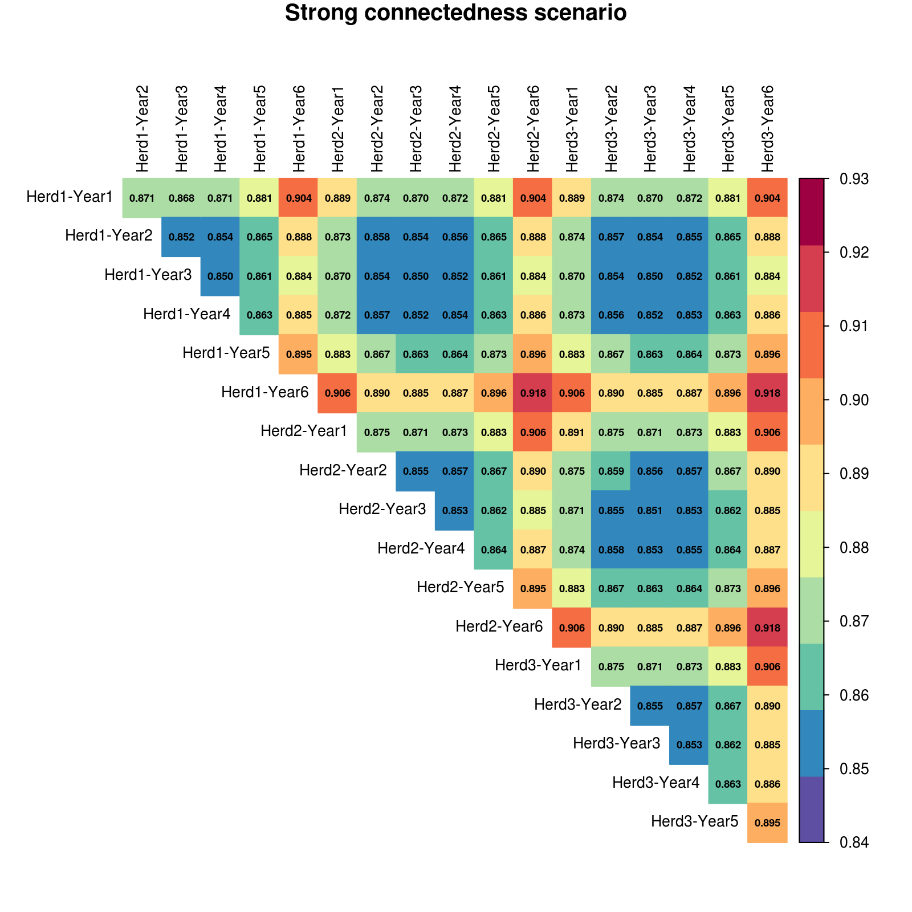


We provide a detailed view of the degree of connectedness based on pairwise prediction error variance of difference (PEVD) values between CGs in two of the simulated scenarios: strong connectedness (SCO; left panel) and weak connectedness (WCO; right panel), from selection year 1 to 6. Note: the PEVD estimates are averages across 20 replicates, with standard deviations ranging from 0.000 and 0.003.

**Figure S2 Differences in PEVD values between connectedness scenarios.**


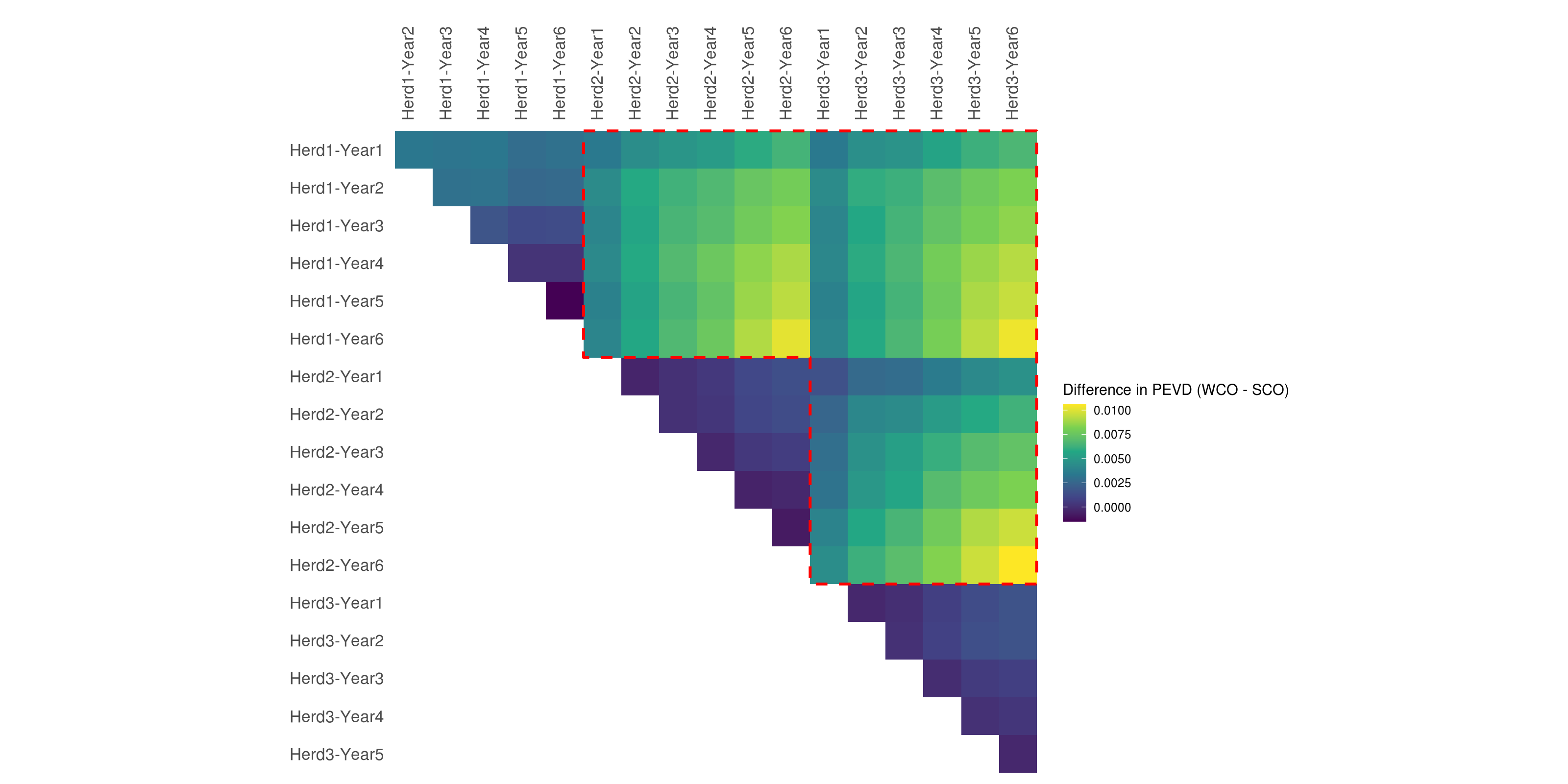


Here we show the difference in the pairwise prediction error variance of difference (PEVD) between the two connectedness scenarios: weak connectedness (WCO) minus strong connectedness (SCO). Red dashed lines delineate the cases where CG comparisons involve pairs of herds across years.

**Figure S3 Connectedness estimates across contemporary groups based on average GDV* for both connectedness scenarios.**


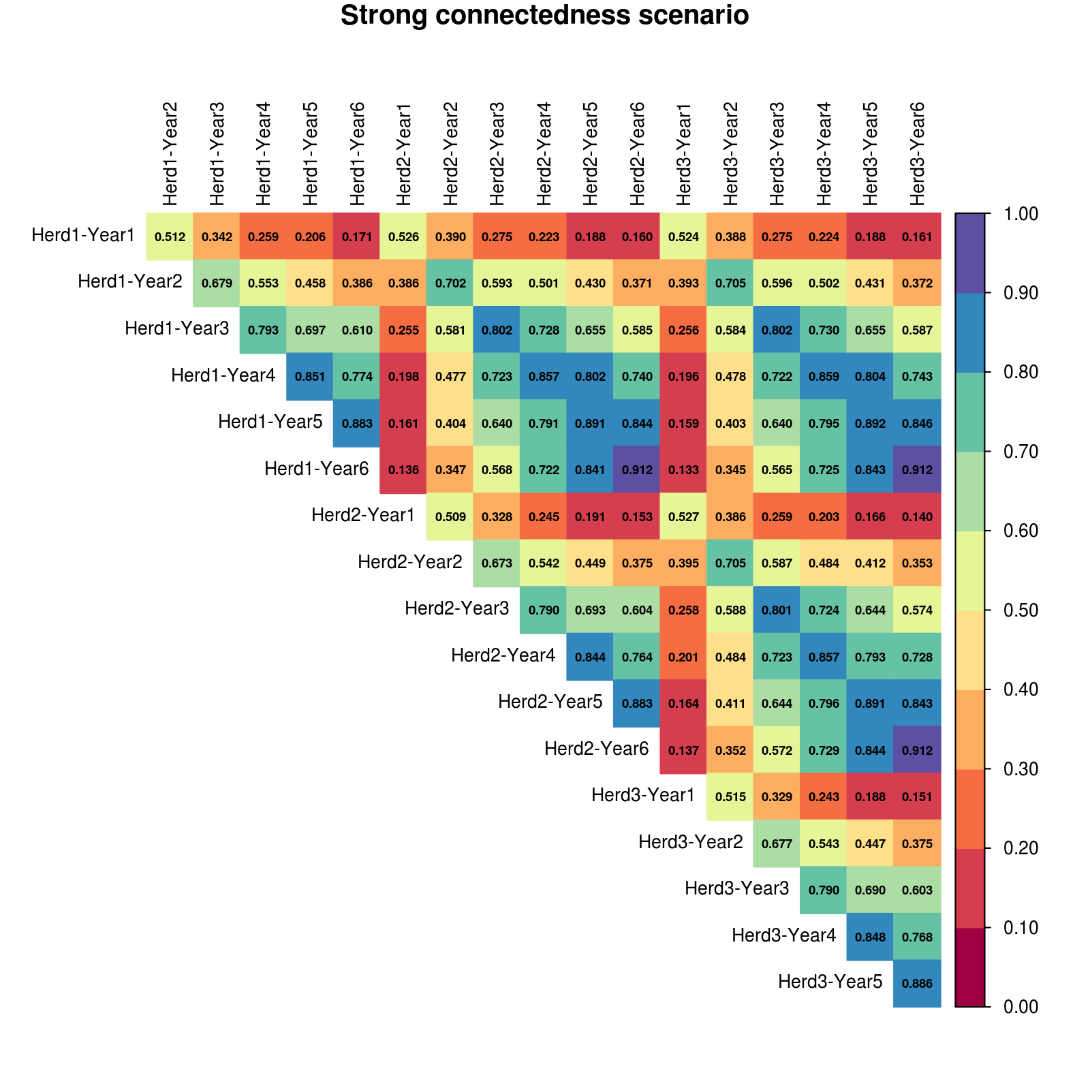

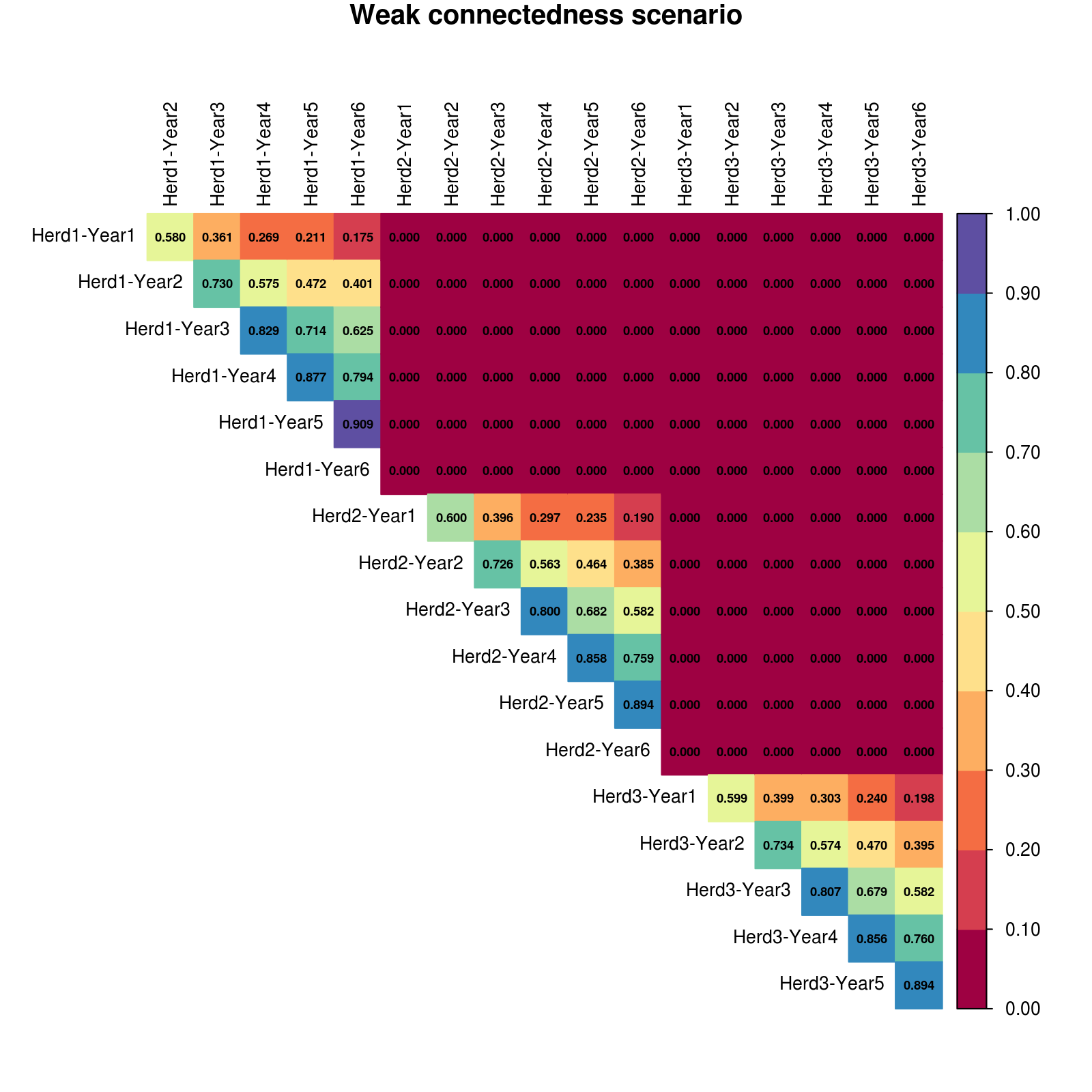


We show the degree of connectedness based on the genetic drift variance (GDV*) values between contemporary groups in two of the simulated scenarios: strong connectedness (SCO; left panel) and weak connectedness (WCO; right panel), from selection year 1 to 6. Note: the GDV* estimates are averages across 20 replicates, with standard deviations ranging from 0.00 to 0.03.

**Figure S4 Common sires across contemporary groups for both connectedness scenarios.**

Here we plot the number of common sires between the different contemporary groups (herd-year) according to the simulated strategy to achieve the two levels of connectedness: strong (right panel) and weak (left panel). Values (colours) represent averages across the 20 replicates. Red dashed lines delineate pairs of herds across years.


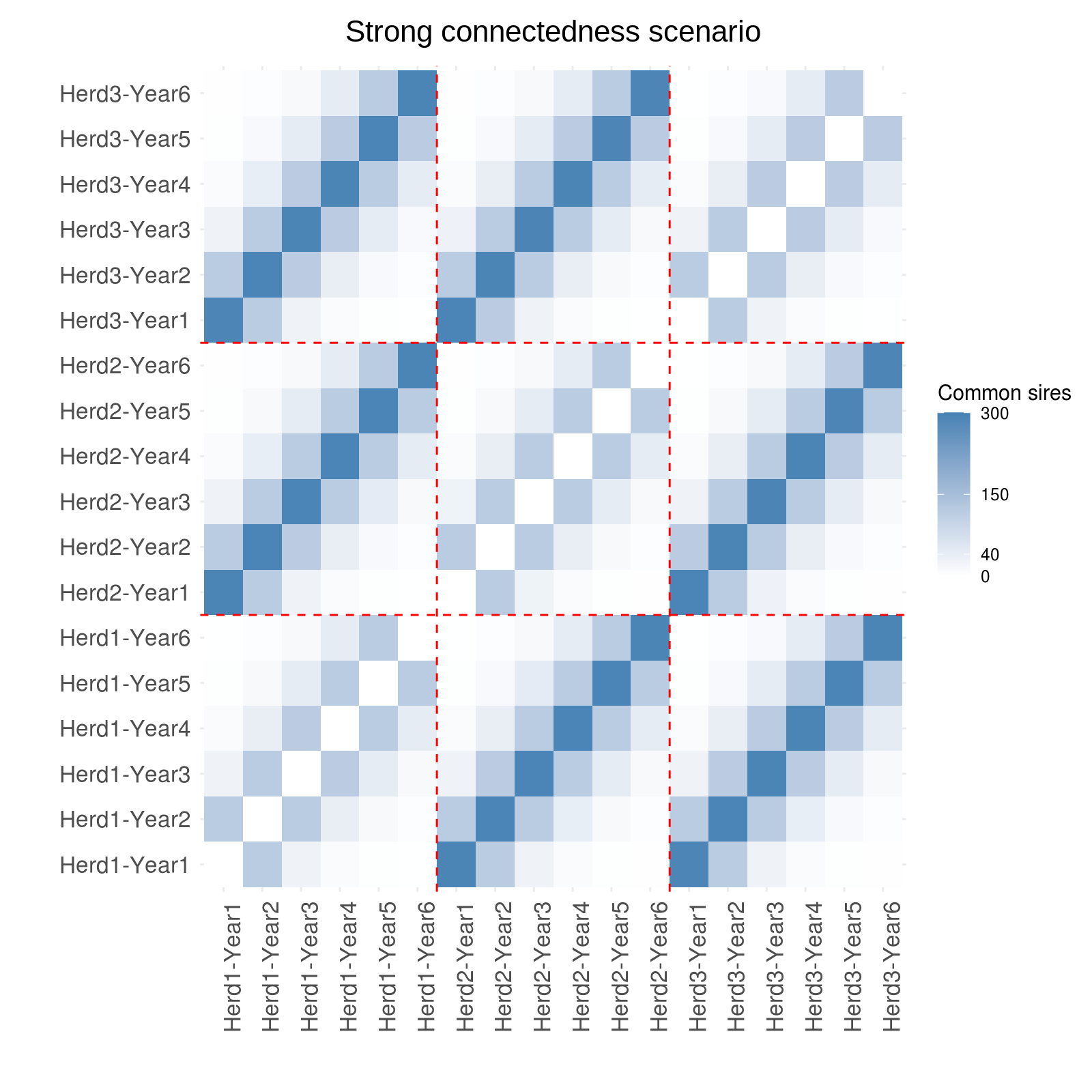

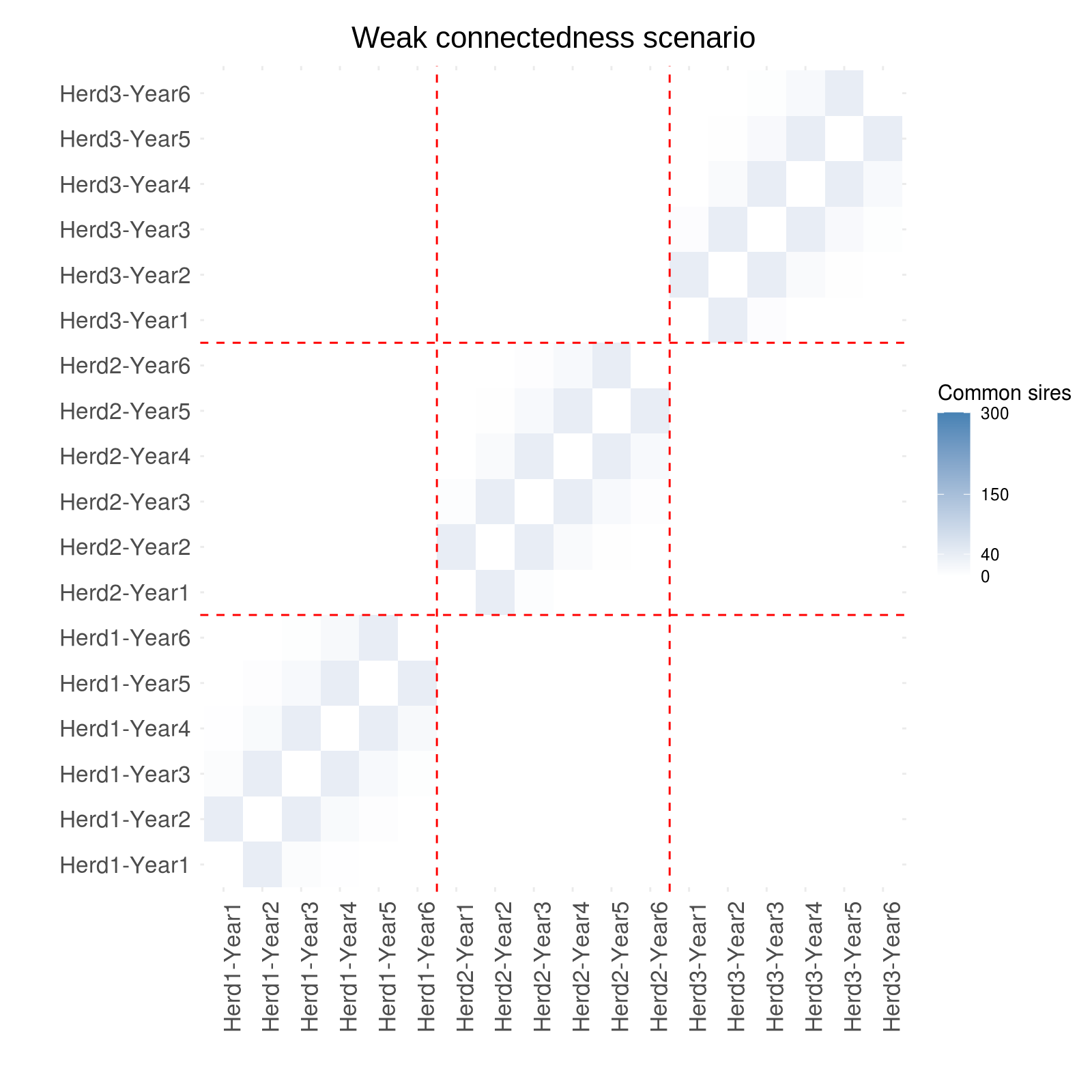


**Figure S5 Plots of the first two principal components (PC) based on the genomic relationship matrix for both connectedness scenarios.**


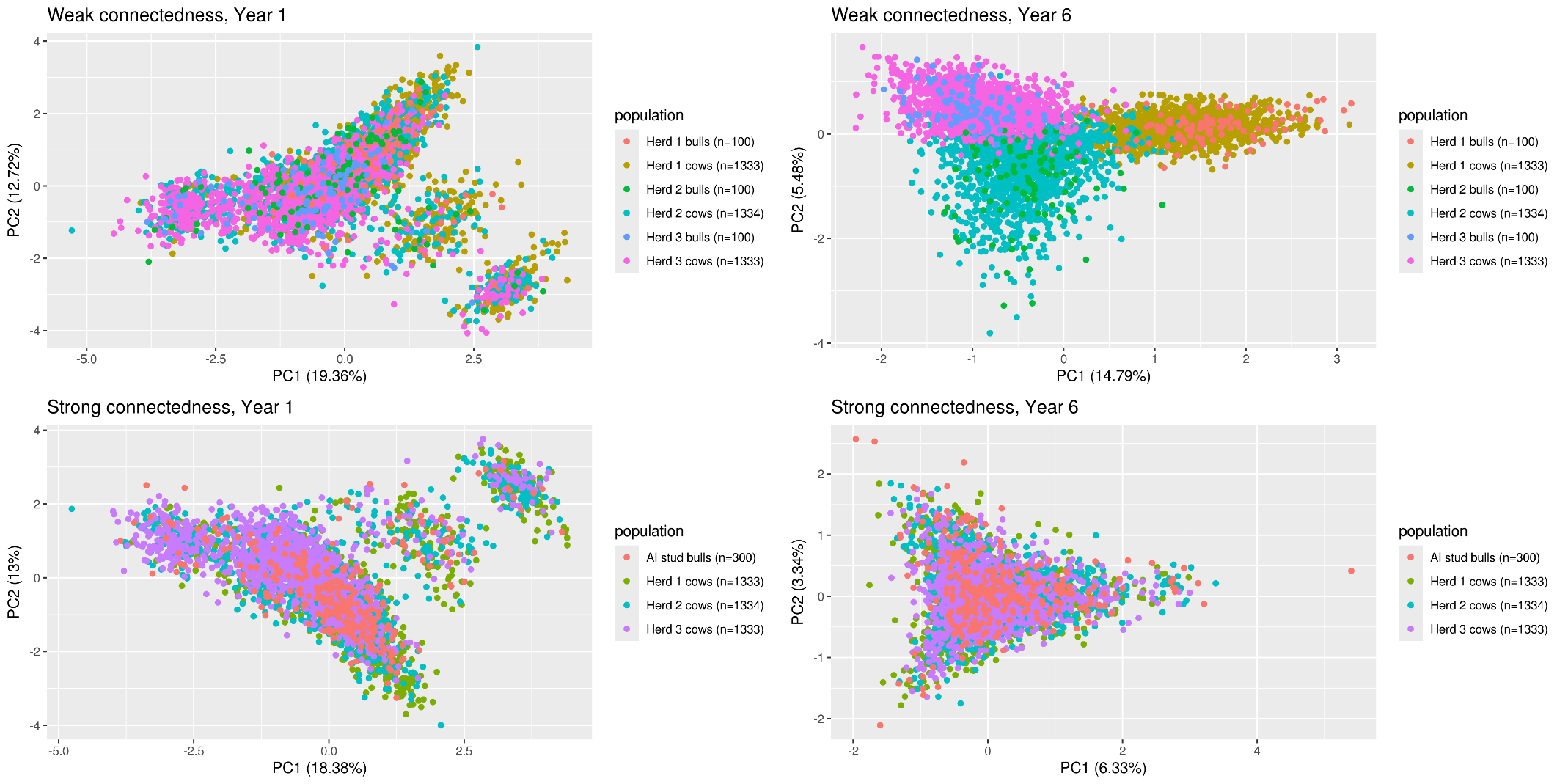


We show the effects of simulated connectedness levels across herds on genomic relationship in selection years 1 and 6 (left and right panels, respectively). After quality control (minor allele frequency > 0.01), the total number of SNPs retained for each PCA ranged from 83,168 to 89,500, depending on the population and the scenario analysed: 4300 animals in both the year one and six of selection for each scenario (WCO and SCO; top and bottom panels, respectively). The PCA was applied to the genomic relationship matrix calculated following VanRaden [41]. Colours within each panel indicate the herds (1 to 3) and sex of animals (cows and bulls). Note: the example was taken from the data of the first replicate.
